# Author Correction: Mapping brain structural differences and neuroreceptor correlates in Parkinson’s disease visual hallucinations

**DOI:** 10.1038/s41467-022-28491-6

**Published:** 2022-02-15

**Authors:** Miriam Vignando, Dominic ffytche, Simon J. G. Lewis, Phil Hyu Lee, Seok Jong Chung, Rimona S. Weil, Michele T. Hu, Clare E. Mackay, Ludovica Griffanti, Delphine Pins, Kathy Dujardin, Renaud Jardri, John-Paul Taylor, Michael Firbank, Grainne McAlonan, Henry K. F. Mak, Shu Leong Ho, Mitul A. Mehta

**Affiliations:** 1 Department of Neuroimaging, King’s College London, Institute of Psychiatry, Psychology and Neuroscience, De Crespigny Park, London, SE5 8AF UK; 2 Department of Old Age Psychiatry, King’s College London, Institute of Psychiatry, Psychology and Neuroscience, De Crespigny Park, London, SE5 8AF UK; 3 ForeFront Parkinson‘s Disease Research Clinic, Brain and Mind Centre, School of Medical Sciences, University of Sydney, Camperdown, NSW Australia; 4 Yonsei University College of Medicine, Seoul, South Korea; 5 Dementia Research Centre, University College London, 8-11 Queen Square, London, WC1M 3BG UK; 6 Wellcome Centre for Neuroimaging, University College London, London, UK; 7 Oxford Parkinson’s Disease Centre, Oxford, UK; 8 Nuffield Department of Clinical Neurosciences, University of Oxford, Oxford, UK; 9 Wellcome Centre for Integrative Neuroimaging, Oxford Centre for Human Brain Activity, Department of Psychiatry, University of Oxford, Oxford, UK; 10 Univ. Lille, Inserm, CHU Lille, U1172 - Centre Lille Neuroscience & Cognition, 59000 Lille, France; 11 Newcastle University, Translational and Clinical Research Institute, Biomedical Research Building, Campus for Ageing and Vitality, Newcastle Upon Tyne, NE4 5PL UK; 12 King’s College London, Institute of Psychiatry, Psychology and Neuroscience, De Crespigny Park, London, SE5 8AF UK; 13 Division of Neurology, Department of Medicine, LKS Faculty of Medicine, University of Hong Kong, Hong Kong, Hong Kong

**Keywords:** Perception, Parkinson's disease

Correction to: *Nature Communications* 10.1038/s41467-022-28087-0, published online 26 January 2022

In the Acknowledgements and Author Contributions sections of this article the grant number relating to King’s College London given for Miriam Vignando was incorrectly given as MR/0059311 and should have been MR/R005931/1. The original article has been corrected.

