# Supplementary material for: Mapping brain structural differences and neuroreceptor correlates in Parkinson’s disease visual hallucinations
Source: Nat Commun. 2022 Jan 26;13:519. doi: 10.1038/s41467-022-28087-0 (PMC8791961; doi:10.1038/s41467-022-28087-0)
Supplement: Supplementary file 1 — Supplementary Information [file 41467_2022_28087_MOESM1_ESM.pdf]

Supplementary References

Supplementary Note 1. The ComBat harmonisation method and its application on this dataset. *p2*

Supplementary Figure 1. Mean thickness before and after harmonisation. *p2*

Supplementary Note 2. Additional information on the full sample. *p3*

Supplementary Note 2a. Meta-analysis on demographics. *P3*

Supplementary Figure 2. Forest plots for Supplementary Note 2a. *p3*

Supplementary Note 2b. Additional Information on the full sample. *p4*

Supplementary Note 3. Results from MANCOVAs. *p5*

Supplementary Table 3a. Results from the main MANCOVAs. *p5*

Supplementary Table 3b. Results from the sensitivity MANCOVAs (N=440 sample). *p7*

Supplementary Table 3c. Results from the 'leave one group out'MANCOVAs. *p9*

Supplementary Note 4. NPI subgroup custom MANCOVAs *p24*

Supplementary Table 4(a). NPI Demographics *p24*

Supplementary Table 4(b). NPI MANCOVA results *p24*

Supplementary Note 5. Ordinal Subgroup analysis *p27*

Supplementary Note 6. Additional models and details about receptor density maps. *p29*

Supplementary Note 6(a). Cortical thickness. *p29*

Supplementary Note 6(b). Volumes. *p29*

Supplementary Figure 3. Additional scatter plots for receptors density profiles. *p29*

Supplementary Note 6(C). Correlations between receptor maps. *P30*

Supplementary Note 7. Principal Component Analysis for cortical thickness *p31*

Supplementary Figure 4. PCA additional details. *p31*

Supplementary Note 8(a). Structural covariance. *p32*

Supplementary Figure5. Correlogram on thickness covariance. *p33*

Supplementary Figure6. Correlogram on surface area covariance. *p34*

Supplementary Figure7. Community plots by lobe. *p35*

Supplementary Note 8(b). Additional network thickness analysis. *p36*

Supplementary Information

Supplementary Note 1. *The ComBat harmonisation method and its application on this dataset.*

As noted in the Methods section, this algorithm was first developed for genomics analyses requiring to pool together data from different datasets (Johnson, Li and Rabinovic, 2007) and has been recently adapted to other measures, such as cortical thickness (Fortin et al., 2018). ComBat applies empirical Bayes (EB) estimation to account for site-specific variance by using an error with a multiplicative scaling factor that is scanner specific. The model allows to retain biological variance, such as that related to age, gender and disease (in our case, hallucinators and non hallucinators), variables that are entered the model as a vector. The algorithm is used on segmented and parcellated data, before running any other statistical analysis and has proven extremely reliable for this type of analyses as described in Fortin et al., 2018 and more recently by Radua et al., 2020. Hierarchical Bayes (HB) is also being proposed to harmonise data from different sites (Kia et al., 2020[1]). However, we chose to use EB and as the purpose of this study was not to assess the suitability of harmonisation models but to build on such harmonisation to develop models for the understanding of the mechanisms underlying visual hallucinations in PD. Secondly, EB is less dependent on the hyper-parameter settings and thus uses data more directly, which for our purposes was the most suitable choice, as HB would possibly be more suitable for approaches whereby a careful specification of hyper-parameters is crucial, as is the case for normative modelling (Kia et al., 2020[1]).

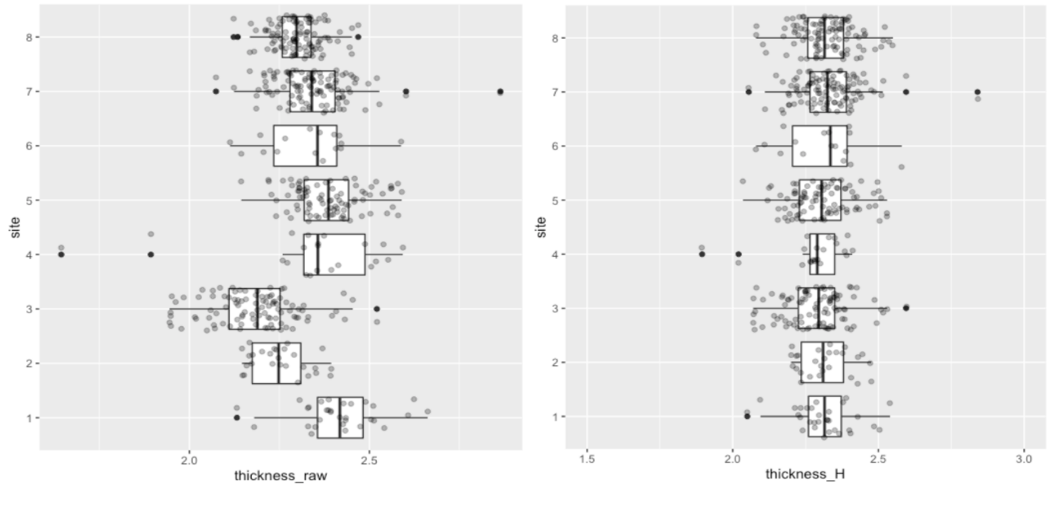

Supplementary Figure 1. *Mean thickness before (thickness_raw) and after harmonisation (thickness_H). On the X axis thickness values, on the Y axis the numbers correspond to the different sites. Sites: 1 = University of Lille, N=31; 2 = Hong Kong University, N=27; 3 = Yonsei University, N=88; 4 = Newcastle University, N=21; 5 = University of Sydney, N =97; 6 = KCL (Dr. ffytche), N= 16; 7 = Oxford Discovery Cohort, N=110; 8 = University College London, N=103. The bounds of the boxes represent the 25th (left edge) and 75th (right edge) percentiles, the center is the median. The whiskers represent the largest (right) and smallest (left) value within 1.5 interquartile range below the 25th percentile (left) or above the 75th (right). The black dots represent outliers that were inspected and retained.*

Supplementary Note 2. *Additional information on the full sample.*

a) *Meta-analysis to determine whether participants in the full sample (N=493) differ on the relevant demographic and clinical variables.*

As we did not have raw data for all groups for all relevant variables, we tested whether our participants were overall matched for age, onset, MMSE, UPDRS and LED by using the mean and standard deviation computed on the raw data, when available, and, when not available, from the data reported in the original publication (note: in some cases the sample was no exactly the same, thus this analysis is an estimate). Most of the groups provided some clinical raw data, but there were missing values for MMSE and UPDRS. We decided to remove those cases from this analysis. The analysis was carried out with R package 'metafor'. Results show that participants are overall matched for age, onset and levodopa equivalent daily dose (LED). Patients differ on MMSE and UPDRS-III scores, with PD-VH presenting lower MMSE and higher UPDRS-III scores (see forest plots and stats below). We excluded the whole Hong Kong Cohort (N=27) because we had only data from the publication about this cohort (except for age). We excluded N = 7 PD-VH and N =15 PD-noVH from the Yonsei study because of UPDRS-III missing data and N= 2 PD-VH, N =15 PD-noVH from the Sydney study because of MMSE missing data.

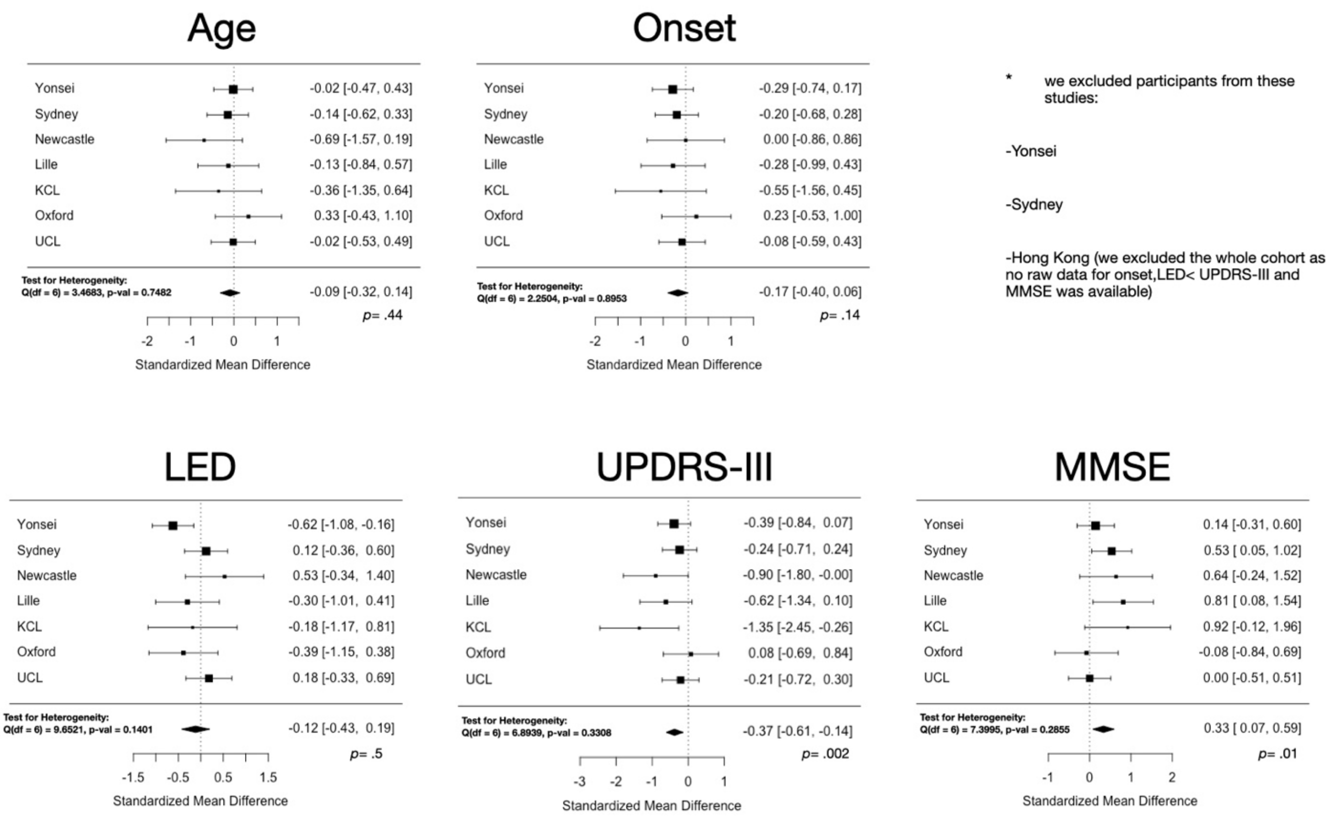

Supplementary Figure 2. The forest plots show a higher UPDRS-III score (difference is 5.8 (sd 2.7) with both groups being within the 'moderate' category as reported in Martínez-Martín et al., 2014[1]) for VH and a lower MMSE (difference

[1] Martínez-Martín, P., Rodríguez-Blázquez, C., Forjaz, M. J., Alvarez-Sánchez, M., Arakaki, T., Bergareche-Yarza, A., ... & Goetz, C. G. (2014). Relationship between the MDS-UPDRS domains and the health-related quality of life of P arkinson's disease patients. *European Journal of Neurology*, *21*(3), 519-524.

is 1 point (sd 0.1; with 29.4 for noVH and 28.8 for VH) score for VH. For each variable and study, we report the mean difference and in the square brackets the confidence intervals.

b) *Information on values for brain volume, total intracranial volume (TIV), total gray matter volume (GM) and age differences in the full sample.* Brain vol. (seg) describes the volume of all voxels in the aparc+aseg.mgz based on which the morphometric information were extracted. This includes voxels that are not background or brainstem, and includes vessel, optic chiasm and CSF segmentations. One-way ANOVAs showed that PD-VH did not significantly differ in TIV from PD-noVH (PD-VH, 1488363.39 ± 192038.64, PD-noVH, 1512273.57 ± 216293.33, p = .84), in the segmented brain volume (PD-VH =0.74 ± 0.05, PD-noVH = 0.74± 0.06, p = .12) but did differ in terms of gray matter volume (PD-VH 559525.16± 63591.98, PD-noVH 584869.68± 71580.08, p = .02).

Supplementary Note 3. *Results from the MANCOVAs.*

Supplementary Table 3(a). *Table for thickness, surface area and volume differences (tests of between-subjects effect and Bonferroni corrected pairwise comparisons) in PD with VH vs. PD no VH patients. Difference was computed with a between subjects (VH vs. noVH) MANCOVA with age, gender and TIV as covariates. The regions entered in this analyses were derived from a VH vs noVH ANOVA, then we FDR corrected the p value of the regions showing difference of the means and retained only those surviving this procedure to be entered in this analysis. Pairwise comparisons are Bonferroni corrected. Regions are reported in descendent order by effect size, as the regions depicted in Figure 1 in the main text are color coded by effect size.*

Region	df	Mean Square	F	p	Partial Eta Squared	Observed Power
R_precuneus	1,492	0.456	16.330	0.000	0.032	0.981
L_octemps_ML.sulc	1,492	0.402	15.570	0.000	0.031	0.976
R_planumtempS	1,492	0.589	14.878	0.000	0.030	0.971
L_sup_par	1,492	0.397	14.624	0.000	0.029	0.968
R_ips	1,492	0.246	13.016	0.000	0.026	0.950
R_parietalS	1,492	0.318	12.189	0.001	0.024	0.936
L_ips	1,492	0.207	11.994	0.001	0.024	0.933
R_occAnt_sul	1,492	0.394	11.724	0.001	0.023	0.927
L_occAnt_sulc	1,492	0.424	11.420	0.001	0.023	0.921
R_supTempsul	1,492	0.203	11.344	0.001	0.023	0.919
L_precuneus	1,492	0.307	10.744	0.001	0.022	0.905
L_ifg_orb	1,492	0.477	10.620	0.001	0.021	0.902
R_occtemp_ML.s	1,492	0.291	10.614	0.001	0.021	0.902
R_insSHort	1,492	0.929	10.431	0.001	0.021	0.897
R_ifgTr	1,492	0.321	10.421	0.001	0.021	0.897
R_frontopolTrs	1,492	0.309	10.217	0.001	0.021	0.891
R_mfg	1,492	0.194	10.107	0.002	0.020	0.887
L_sfg	1,492	0.229	9.913	0.002	0.020	0.881
L_ITG	1,492	0.338	9.699	0.002	0.019	0.875
L_cingMarg_s	1,492	0.240	9.647	0.002	0.019	0.873
L_ifg_op	1,492	0.234	9.454	0.002	0.019	0.866
L_inf_angular	1,492	0.244	9.385	0.002	0.019	0.864
R_precentrInf	1,492	0.230	9.248	0.002	0.019	0.859
L_temp_pole	1,492	0.506	9.193	0.003	0.018	0.857
R_subcall	1,492	1.222	8.688	0.003	0.017	0.837
L_cuneus	1,492	0.159	8.579	0.004	0.017	0.832
R_cuneus	1,492	0.164	8.030	0.005	0.016	0.807
L_parietoOc	1,492	0.156	7.819	0.005	0.016	0.797
R_occsupG	1,492	0.209	7.230	0.007	0.015	0.765
R_octemp_ML	1,492	0.123	7.086	0.008	0.014	0.757
R_supFr	1,492	0.134	7.000	0.008	0.014	0.752
R_latFissureP	1,492	0.152	6.977	0.009	0.014	0.751
R_sfg	1,492	0.144	6.728	0.010	0.014	0.735
R_ifg_op	1,492	0.202	6.680	0.010	0.014	0.732
R_mtg	1,492	0.218	6.541	0.011	0.013	0.723

R_infang	1,492	0.153	6.391	0.012	0.013	0.713
R_subpar	1,492	0.203	6.344	0.012	0.013	0.710
L_stg_lat	1,492	0.276	6.341	0.012	0.013	0.710
L_inf_smg	1,492	0.164	6.317	0.012	0.013	0.708
R_itg	1,492	0.207	6.241	0.013	0.013	0.703
L_occ_s	1,492	0.198	6.197	0.013	0.013	0.700
L_plan_tempor_s	1,492	0.249	6.173	0.013	0.012	0.698
L_rectus	1,492	0.203	6.007	0.015	0.012	0.687
R_temporalPOle	1,492	0.357	5.977	0.015	0.012	0.684
L_stg	1,492	0.299	5.682	0.018	0.012	0.662
R_latfusif	1,492	0.235	5.606	0.018	0.011	0.657
L_ifg_triang	1,492	0.163	5.578	0.019	0.011	0.654
L_ifs	1,492	0.098	5.560	0.019	0.011	0.653
R_orbG	1,492	0.149	5.499	0.019	0.011	0.648
R_precentr	1,492	0.316	5.472	0.020	0.011	0.646
R_ifg_orb	1,492	0.301	5.354	0.021	0.011	0.637
R_precentS	1,492	0.159	5.248	0.022	0.011	0.628
L_precentralg	1,492	0.285	5.203	0.023	0.011	0.624
L_mfg	1,492	0.099	5.038	0.025	0.010	0.610
R_moccG	1,492	0.125	5.016	0.026	0.010	0.608
L_paracentral_g	1,492	0.197	4.893	0.027	0.010	0.598
R_rectus	1,492	0.206	4.810	0.029	0.010	0.591
L_fiss_hor_ant	1,492	0.270	4.777	0.029	0.010	0.588
L_fis_post	1,492	0.112	4.758	0.030	0.010	0.586
L_occ_m	1,492	0.116	4.718	0.030	0.010	0.582
R_supTrans	1,492	0.103	4.441	0.036	0.009	0.557
L_sfs	1,492	0.090	4.356	0.037	0.009	0.549
R_stglat	1,492	0.187	4.276	0.039	0.009	0.541
L_octempSul_lat	1,492	0.158	4.263	0.039	0.009	0.540
R_octemp_Lat	1,492	0.162	4.205	0.041	0.009	0.535
L_trsv	1,492	0.096	4.195	0.041	0.009	0.534
L_collaTr_ants	1,492	0.186	4.184	0.041	0.009	0.533
L_central	1,492	0.070	4.100	0.043	0.008	0.524
L_ins_g_short	1,492	0.298	4.068	0.044	0.008	0.521
R_inffrontsulc	1,492	0.069	3.883	0.049	0.008	0.503
R_paracentr	1,492	0.152	3.872	0.050	0.008	0.502
R_latfissAntvert	1,492	0.320	3.836	0.051	0.008	0.498
R_parietoOcc	1,492	0.067	3.724	0.054	0.008	0.486

Surface area ANCOVA (age, gender, TIV as covariates)

Region	*mean square*	*df*	*F*	*partial eta2*	*p*	*obs. power*
R_occtemp. gyrus	796730.83	1,488	10.876	0.022	0.001	0.908

Subcortical volumes MANCOVA (age, gender and TIV as covariates)

Region	*mean square*	*df*	*F*	*partial eta2*	*p*	*obs. power*

Left-Cerebellum-WM	25136509.5	1488	4.80338071	0.011	0.029	0.59
R amygdala	278036.6	1488	5.33541167	0.021	0.021	0.635
L amygdala	349741.74	1488	6.66409361	0.013	0.01	0.731

Supplementary Table 3(b). *Additional MANCOVA (VH vs noVH) with age, gender and TIV (as for the main model in (a)) and onset, LED, MMSE, UPDRS-III as covariates on a sample of N=440 participants (121 VH) for whom we had this additional data. A few participants had missing data that were filled with the mean of the group (*N = 7 PD-VH and N =15 PD-noVH from the Yonsei study and N= 2 PD-VH, N =15 PD-noVH from the Sydney study). For cortical thickness there was also a significant main effect of age [$F(88,344)=2.38$, $p<.001$)], gender [$F(88,344)=1.56$, $p = .003$)], TIV [$F(88,344)=3.79$ $p<.001$)], but not of LED, onset, MMSE, UPDRS-III.

Cortical thickness (cov. Age, gender, TIV, onset, LED, MMSE, UPDRS-III)

Region	*mean square*	*df*	*F*	*partial eta2*	*p*	*obs. power*
R_ips	0,320	1,439	17,009	0,038	0,000	0,984
L_octemps_ML	0,350	1,439	13,550	0,030	0,000	0,957
R_planumtempS	0,496	1,439	12,879	0,029	0,000	0,947
L_ips	0,222	1,439	12,692	0,029	0,000	0,945
R_precuneus	0,350	1,439	12,409	0,028	0,000	0,940
L_sup_par	0,342	1,439	12,355	0,028	0,000	0,939
L_occAnt_sulc	0,428	1,439	11,559	0,026	0,001	0,924
L_ITG	0,385	1,439	11,136	0,025	0,001	0,915
L_cingMarg_s	0,272	1,439	10,855	0,025	0,001	0,908
R_mfg	0,203	1,439	10,795	0,024	0,001	0,906
R_supTempsul	0,191	1,439	10,765	0,024	0,001	0,906
L_temp_pole	0,575	1,439	10,425	0,024	0,001	0,896
R_parietalS	0,269	1,439	10,375	0,024	0,001	0,895
L_precuneus	0,287	1,439	9,875	0,022	0,002	0,880
L_ifg_op	0,241	1,439	9,672	0,022	0,002	0,874
R_frontopolTrs	0,298	1,439	9,599	0,022	0,002	0,871
R_precentrInf	0,235	1,439	9,353	0,021	0,002	0,862
R_cuneus	0,197	1,439	9,128	0,021	0,003	0,854
R_occAnt_sul	0,308	1,439	9,123	0,021	0,003	0,854
R_precentS	0,270	1,439	8,994	0,020	0,003	0,849
R_occsupG	0,252	1,439	8,842	0,020	0,003	0,843
L_ifg_orb	0,396	1,439	8,642	0,020	0,003	0,835
R_subpar	0,257	1,439	8,081	0,018	0,005	0,810
R_supFr	0,152	1,439	8,063	0,018	0,005	0,809
R_infang	0,182	1,439	7,742	0,018	0,006	0,793
L_ifs	0,137	1,439	7,733	0,018	0,006	0,792
L_inf_angular	0,199	1,439	7,652	0,017	0,006	0,788
L_cuneus	0,141	1,439	7,342	0,017	0,007	0,771
L_sfg	0,167	1,439	7,312	0,017	0,007	0,770
R_ifgTr	0,220	1,439	7,191	0,016	0,008	0,763
R_occtemp_ML.s	0,191	1,439	6,843	0,016	0,009	0,742

L_parietoOc	0,140	1,439	6,816	0,016	0,009	0,741
L_fiss_hor_ant	0,372	1,439	6,781	0,015	0,010	0,738
R_latFissureP	0,146	1,439	6,765	0,015	0,010	0,737
L_mfg	0,127	1,439	6,386	0,015	0,012	0,713
L_paracentral_g	0,248	1,439	6,283	0,014	0,013	0,706
R_Jenses	0,257	1,439	6,072	0,014	0,014	0,691
R_mtg	0,202	1,439	6,057	0,014	0,014	0,690
R_ifg_op	0,173	1,439	5,794	0,013	0,016	0,671
L_occ_s	0,177	1,439	5,672	0,013	0,018	0,661
R_paracentr	0,218	1,439	5,603	0,013	0,018	0,656
R_supTrans	0,132	1,439	5,562	0,013	0,019	0,653
L_plan_tempor_s	0,223	1,439	5,557	0,013	0,019	0,653
R_transpost	0,224	1,439	5,425	0,012	0,020	0,642
L_collaTr_ants	0,235	1,439	5,415	0,012	0,020	0,641
L_ifg_triang	0,158	1,439	5,349	0,012	0,021	0,636
R_temporalPOle	0,311	1,439	5,347	0,012	0,021	0,636
R_ifg_orb	0,294	1,439	5,161	0,012	0,024	0,621
R_insSHort	0,455	1,439	5,083	0,012	0,025	0,614
L_rectus	0,175	1,439	5,013	0,011	0,026	0,608
R_sfg	0,104	1,439	4,976	0,011	0,026	0,605
R_precentr	0,279	1,439	4,942	0,011	0,027	0,602
L_inf_smg	0,129	1,439	4,926	0,011	0,027	0,600
R_octemp_ML	0,088	1,439	4,916	0,011	0,027	0,600
R_itg	0,164	1,439	4,892	0,011	0,027	0,598
L_fis_ver_ant	0,264	1,439	4,810	0,011	0,029	0,590
R_latfissAntvert	0,411	1,439	4,796	0,011	0,029	0,589
R_moccG	0,118	1,439	4,794	0,011	0,029	0,589
R_inffrontsulc	0,080	1,439	4,521	0,010	0,034	0,564
R_subcall	0,617	1,439	4,423	0,010	0,036	0,555
R_octemp_Lat	0,170	1,439	4,310	0,010	0,038	0,544
L_sfs	0,087	1,439	4,271	0,010	0,039	0,541
L_infTempSul	0,125	1,439	4,244	0,010	0,040	0,538
R_latfusif	0,177	1,439	4,110	0,009	0,043	0,525
L_trsv	0,093	1,439	4,017	0,009	0,046	0,516
R_rectus	0,176	1,439	4,015	0,009	0,046	0,516
L_precentralg	0,205	1,439	3,804	0,009	0,052	0,494
L_precentrInf	0,099	1,439	3,694	0,008	0,055	0,483
R_INfparsupramarg	0,097	1,439	3,532	0,008	0,061	0,466

Surface area ANCOVA (cov. Age, gender, TIV, onset, LED, MMSE, UPDRS-III)

Region	*mean square*	*df*	*F*	*partial eta2*	*p*	*obs. power*
R_occtemp. gyrus	519216.46	1,439	7.123	0.02	0.008	0.76

For subcortical volume there was also a significant main effect of age [F(1,439)=71.23, p <.001)], TIV [F(1,439)=27.71, p <.001)] and onset [F(1,439)=4.67 p = .03)] but not of MMSE, UPDRS-III or LED.

Subcortical volumes MANCOVA (age, gender and TIV as covariates)

Region	*mean square*	*df*	*F*	*partial eta2*	*p*	*obs. power*
R amygdala	251688.77	1,439	4.996	0.011	0.026	0.61
L amygdala	321003.61	1,439	6.310	0.012	0.012	0.71
R caudate *	2041364.73	1,439	7.068	0.016	0.008	0.756

*The caudate is bigger in VH than noVH (as in the second subgroup analysis reported in S4).

For subcortical volume there was also a significant main effect of age [$F(16,416)=14.13$, $p <.001$)], gender [$F(16,416)=1.87$, $p = .02$)], TIV [$F(16,416)=32.55$, $p <.001$)] and LED [$F(16,416)=1.72$, $p = .04$)] but not of MMSE, UPDRS-III or disease onset.

.

Supplementary Table 3(c). *Tables for the 'leave one group out' approach on the full sample, with age, gender and TIV as covariates. We report tests of between-subjects effect and Bonferroni corrected pairwise comparisons in PD with VH vs. PD no VH patients. The regions entered in this analyses were derived from a VH vs noVH ANOVA, then we FDR corrected the p value of the regions showing difference of the means and retained only those surviving this procedure to be entered in this analysis. Pairwise comparisons are Bonferroni corrected. Regions are reported in descendent order by effect size.*

For thickness we observe throughout consistently results, with a variable reduction of the regions found when not including the Yonsei, Newcastle, Sydney and UCL groups in particular in the lateral fissures and collateral transverse and paracentral regions.

Cortical thickness MANCOVA (cov: age, gender, TIV) - leave one group out test

without Lille group

Region	Mean Square	F	Sig.	Partial Eta Squared	Power
R_precuneus	0.407	14.530	0.000	0.031	0.967
R_planumtempS	0.525	13.150	0.000	0.028	0.951
L_sup_par	0.346	12.781	0.000	0.027	0.946
R_ips	0.223	11.723	0.001	0.025	0.927
L_octemps_ML	0.296	11.483	0.001	0.025	0.922
L_ITG	0.394	11.392	0.001	0.024	0.920
R_mfg	0.213	11.309	0.001	0.024	0.919
R_parietalS	0.292	11.119	0.001	0.024	0.914
L_occAnt_sulc	0.420	11.076	0.001	0.024	0.913
L_ips	0.186	10.786	0.001	0.023	0.906
L_ifg_op	0.259	10.505	0.001	0.022	0.899
L_sfg	0.236	10.258	0.001	0.022	0.892
R_subcall	1.450	10.182	0.002	0.022	0.890

R_frontopolTrs	0.308	10.088	0.002	0.022	0.887
L_precuneus	0.285	9.968	0.002	0.021	0.883
L_inf_angular	0.243	9.481	0.002	0.020	0.867
R_supTempsul	0.171	9.461	0.002	0.020	0.866
L_ifg_orb	0.409	9.093	0.003	0.020	0.853
R_occAnt_sul	0.302	9.031	0.003	0.019	0.851
R_precentrInf	0.226	9.027	0.003	0.019	0.850
R_ifgTr	0.267	8.736	0.003	0.019	0.839
R_supFr	0.162	8.483	0.004	0.018	0.828
L_cuneus	0.157	8.460	0.004	0.018	0.827
R_occtemp_ML.s	0.228	8.342	0.004	0.018	0.822
R_sfg	0.171	8.045	0.005	0.017	0.808
R_insSHort	0.697	7.800	0.005	0.017	0.796
R_itg	0.258	7.783	0.005	0.017	0.795
L_cingMarg_s	0.196	7.697	0.006	0.017	0.791
R_precentS	0.231	7.509	0.006	0.016	0.781
L_temp_pole	0.409	7.409	0.007	0.016	0.775
L_plan_tempor_s	0.281	7.043	0.008	0.015	0.754
L_occ_s	0.223	6.989	0.008	0.015	0.751
L_inf_smg	0.172	6.693	0.010	0.014	0.733
L_stg_lat	0.274	6.344	0.012	0.014	0.710
L_parietoOc	0.122	6.120	0.014	0.013	0.695
R_paracentr	0.240	6.100	0.014	0.013	0.693
R_latFissureP	0.133	6.063	0.014	0.013	0.690
R_latfusif	0.251	5.936	0.015	0.013	0.681
R_octemp_ML	0.101	5.896	0.016	0.013	0.678
R_precentr	0.333	5.794	0.016	0.013	0.671
R_ifg_op	0.170	5.654	0.018	0.012	0.660
R_mtg	0.189	5.642	0.018	0.012	0.659
R_occsupG	0.157	5.399	0.021	0.012	0.640
R_cuneus	0.109	5.382	0.021	0.012	0.639
R_temporalPOle	0.316	5.285	0.022	0.011	0.631
L_precentralg	0.284	5.253	0.022	0.011	0.628
R_infang	0.123	5.155	0.024	0.011	0.620
L_mfg	0.100	5.104	0.024	0.011	0.616
R_rectus	0.216	5.081	0.025	0.011	0.614
R_subpar	0.162	5.031	0.025	0.011	0.610
L_octempSul_lat	0.186	4.963	0.026	0.011	0.604
L_paracentral_g	0.199	4.954	0.027	0.011	0.603
L_fiss_hor_ant	0.274	4.839	0.028	0.010	0.593
L_occ_m	0.116	4.770	0.029	0.010	0.587
R_orbG	0.129	4.748	0.030	0.010	0.585
L_infTempSul	0.139	4.687	0.031	0.010	0.579
L_fis_post	0.110	4.680	0.031	0.010	0.579
L_stg	0.235	4.506	0.034	0.010	0.563

L_trsv	0.104	4.481	0.035	0.010	0.561
L_insulaA	0.217	4.460	0.035	0.010	0.559
L_fis_ver_ant	0.251	4.435	0.036	0.010	0.556
L_sfs	0.089	4.310	0.038	0.009	0.544
R_stglat	0.183	4.208	0.041	0.009	0.535
L_ifs	0.072	4.059	0.045	0.009	0.520
L_ifg_triang	0.114	3.943	0.048	0.009	0.509
L_rectus	0.129	3.823	0.051	0.008	0.497
R_ifg_orb	0.211	3.785	0.052	0.008	0.493
R_latfissAntvert	0.317	3.746	0.054	0.008	0.489
L_occtemp_Ling	0.058	3.720	0.054	0.008	0.486

without Yonsei group

Region	Mean Square	F	Sig.	Partial Eta Squared	Power
R_insSHort	1.359	15.244	0.000	0.038	0.973
R_precuneus	0.344	12.156	0.001	0.030	0.935
L_octemps_ML	0.305	11.836	0.001	0.029	0.929
L_ifg_orb	0.501	11.266	0.001	0.028	0.917
R_occAnt_sul	0.336	9.765	0.002	0.024	0.876
L_occAnt_sulc	0.326	8.862	0.003	0.022	0.844
L_stg_lat	0.383	8.787	0.003	0.022	0.841
L_sup_par	0.224	8.336	0.004	0.021	0.821
L_sfg	0.182	7.824	0.005	0.020	0.797
R_octemp_ML	0.141	7.796	0.005	0.020	0.795
R_occtemp_ML.s	0.214	7.733	0.006	0.019	0.792
R_planumtempS	0.309	7.668	0.006	0.019	0.789
R_occsupG	0.215	7.617	0.006	0.019	0.786
L_ips	0.126	7.396	0.007	0.019	0.774
R_sfg	0.159	7.362	0.007	0.018	0.772
R_ips	0.131	6.944	0.009	0.017	0.748
R_ifgTr	0.212	6.868	0.009	0.017	0.744
L_precuneus	0.196	6.822	0.009	0.017	0.741
L_inf_angular	0.170	6.570	0.011	0.017	0.725
R_cuneus	0.136	6.460	0.011	0.016	0.718
L_ifg_op	0.159	6.417	0.012	0.016	0.715
R_rectus	0.280	6.408	0.012	0.016	0.714
L_temp_pole	0.347	6.206	0.013	0.016	0.700
R_orbG	0.167	6.035	0.014	0.015	0.688
L_plan_tempor_s	0.243	6.025	0.015	0.015	0.687
R_parietalS	0.154	5.933	0.015	0.015	0.681
R_frontopolTrs	0.169	5.799	0.017	0.015	0.671
L_parietoOc	0.116	5.790	0.017	0.015	0.670
L_rectus	0.202	5.775	0.017	0.015	0.669
L_ITG	0.205	5.753	0.017	0.015	0.667

R_precentS	0.169	5.706	0.017	0.014	0.664
L_cuneus	0.106	5.652	0.018	0.014	0.660
R_temporalPOle	0.334	5.553	0.019	0.014	0.652
L_ins_g_short	0.414	5.534	0.019	0.014	0.651
R_subpar	0.178	5.525	0.019	0.014	0.650
R_precentrInf	0.134	5.428	0.020	0.014	0.642
R_supTempsul	0.095	5.361	0.021	0.014	0.637
R_stglat	0.233	5.248	0.022	0.013	0.628
R_mfg	0.099	5.203	0.023	0.013	0.624
L_mfg	0.100	5.086	0.025	0.013	0.614
R_infang	0.124	5.027	0.026	0.013	0.609
R_subcall	0.723	4.995	0.026	0.013	0.606
R_supFr	0.092	4.975	0.026	0.013	0.604
R_latfusif	0.199	4.867	0.028	0.012	0.595
L_cingMarg_s	0.111	4.604	0.033	0.012	0.572
L_fis_post	0.111	4.602	0.033	0.012	0.571
L_inf_smg	0.119	4.535	0.034	0.011	0.565
L_stg	0.240	4.438	0.036	0.011	0.556
R_ifg_orb	0.240	4.326	0.038	0.011	0.546
R_latFissureP	0.092	4.205	0.041	0.011	0.534
L_sfs	0.077	3.894	0.049	0.010	0.503
L_trsv	0.088	3.883	0.049	0.010	0.502

without Newcastle group

Region	Mean Square	F	Sig.	Partial Eta Squared	Power
R_precuneus	0.418	14.954	0.000	0.031	0.971
L_sup_par	0.410	14.920	0.000	0.031	0.971
R_ips	0.246	12.921	0.000	0.027	0.948
R_planumtempS	0.511	12.817	0.000	0.027	0.947
R_parietalS	0.334	12.620	0.000	0.026	0.944
L_ips	0.200	11.599	0.001	0.024	0.925
L_octemps_ML	0.290	11.309	0.001	0.024	0.919
L_occAnt_sulc	0.419	11.291	0.001	0.024	0.918
R_ifgTr	0.300	9.768	0.002	0.020	0.877
L_precuneus	0.269	9.441	0.002	0.020	0.866
L_cuneus	0.171	9.172	0.003	0.019	0.856
R_occAnt_sul	0.300	8.967	0.003	0.019	0.848
R_mfg	0.171	8.959	0.003	0.019	0.848
L_ifg_op	0.219	8.867	0.003	0.019	0.844
R_supTempsul	0.154	8.715	0.003	0.018	0.838
R_frontopolTrs	0.267	8.678	0.003	0.018	0.836
L_cingMarg_s	0.216	8.625	0.003	0.018	0.834
L_sfg	0.197	8.501	0.004	0.018	0.829
L_inf_angular	0.221	8.413	0.004	0.018	0.825

L_ifg_orb	0.363	8.077	0.005	0.017	0.810
R_insSHort	0.711	7.999	0.005	0.017	0.806
R_precentrInf	0.195	7.910	0.005	0.017	0.801
L_ITG	0.274	7.847	0.005	0.017	0.798
R_occtemp_ML.s	0.206	7.525	0.006	0.016	0.782
R_occsupG	0.219	7.442	0.007	0.016	0.777
L_temp_pole	0.396	7.142	0.008	0.015	0.760
L_occ_s	0.232	7.128	0.008	0.015	0.759
R_cuneus	0.140	6.820	0.009	0.014	0.741
R_subcall	0.918	6.433	0.012	0.014	0.716
L_parietoOc	0.127	6.382	0.012	0.013	0.713
R_supFr	0.120	6.275	0.013	0.013	0.705
R_octemp_ML	0.106	6.091	0.014	0.013	0.692
R_ifg_op	0.179	5.926	0.015	0.013	0.681
L_stg_lat	0.257	5.901	0.016	0.012	0.679
R_infang	0.135	5.562	0.019	0.012	0.653
R_sfg	0.118	5.521	0.019	0.012	0.650
L_ifs	0.093	5.372	0.021	0.011	0.638
L_ifg_triang	0.156	5.316	0.022	0.011	0.633
R_latFissureP	0.111	5.110	0.024	0.011	0.616
R_precentr	0.294	5.102	0.024	0.011	0.616
L_plan_tempor_s	0.203	5.053	0.025	0.011	0.612
L_stg	0.257	4.892	0.027	0.010	0.598
L_rectus	0.166	4.876	0.028	0.010	0.596
R_precentS	0.141	4.663	0.031	0.010	0.577
L_inf_smg	0.122	4.652	0.032	0.010	0.576
L_mfg	0.088	4.477	0.035	0.009	0.560
R_mtg	0.143	4.330	0.038	0.009	0.546
R_subpar	0.137	4.313	0.038	0.009	0.545
R_moccG	0.107	4.281	0.039	0.009	0.542
R_orbG	0.114	4.225	0.040	0.009	0.536
R_itg	0.138	4.183	0.041	0.009	0.532
R_latfusif	0.166	4.014	0.046	0.009	0.516
L_precentralg	0.217	3.964	0.047	0.008	0.511
L_paracentral_g	0.158	3.932	0.048	0.008	0.508
L_sfs	0.076	3.717	0.054	0.008	0.486
L_trsv	0.085	3.714	0.055	0.008	0.485

without KCL group

Region	Mean Square	F	Sig.	Partial Eta Squared	Power
R_precuneus	0.448	16.069	0.000	0.033	0.979
L_octemps_ML	0.377	14.677	0.000	0.030	0.969
L_sup_par	0.364	13.440	0.000	0.028	0.955

R_planumtempS	0.514	12.895	0.000	0.027	0.948
R_ips	0.234	12.359	0.000	0.026	0.939
L_ips	0.198	11.450	0.001	0.024	0.922
R_occAnt_sul	0.377	11.295	0.001	0.023	0.918
R_parietalS	0.288	11.077	0.001	0.023	0.913
R_frontopolTrs	0.333	10.939	0.001	0.023	0.910
L_ifg_orb	0.465	10.292	0.001	0.021	0.893
L_occAnt_sulc	0.376	10.150	0.002	0.021	0.889
L_precuneus	0.290	10.133	0.002	0.021	0.888
R_occtemp_ML.s	0.276	10.042	0.002	0.021	0.885
L_ITG	0.332	9.648	0.002	0.020	0.873
L_temp_pole	0.509	9.257	0.002	0.019	0.859
R_supTempsul	0.163	9.155	0.003	0.019	0.855
L_sfg	0.210	9.130	0.003	0.019	0.854
R_mfg	0.168	8.801	0.003	0.018	0.842
R_ifgTr	0.271	8.793	0.003	0.018	0.841
R_precentrInf	0.223	8.769	0.003	0.018	0.840
L_ifg_op	0.217	8.753	0.003	0.018	0.840
L_inf_angular	0.227	8.749	0.003	0.018	0.839
L_cingMarg_s	0.208	8.368	0.004	0.017	0.823
L_parietoOc	0.168	8.293	0.004	0.017	0.820
R_subcall	1.109	7.847	0.005	0.016	0.798
R_cuneus	0.161	7.816	0.005	0.016	0.797
R_subpar	0.250	7.801	0.005	0.016	0.796
L_cuneus	0.142	7.665	0.006	0.016	0.789
R_insSHort	0.665	7.522	0.006	0.016	0.781
R_occsupG	0.213	7.395	0.007	0.015	0.775
L_rectus	0.230	6.782	0.009	0.014	0.739
R_supFr	0.126	6.543	0.011	0.014	0.723
R_sfg	0.139	6.517	0.011	0.014	0.722
R_mtg	0.200	6.044	0.014	0.013	0.689
R_latFissureP	0.131	5.965	0.015	0.012	0.683
R_ifg_orb	0.317	5.669	0.018	0.012	0.661
R_octemp_ML	0.099	5.611	0.018	0.012	0.657
L_precentralg	0.302	5.555	0.019	0.012	0.653
L_paracentral_g	0.218	5.443	0.020	0.011	0.644
R_precentr	0.314	5.437	0.020	0.011	0.643
R_infang	0.128	5.392	0.021	0.011	0.640
R_itg	0.176	5.327	0.021	0.011	0.634
L_inf_smg	0.137	5.313	0.022	0.011	0.633
R_ifg_op	0.161	5.300	0.022	0.011	0.632
L_ifg_triang	0.153	5.201	0.023	0.011	0.624
L_ifs	0.092	5.201	0.023	0.011	0.624
L_occ_s	0.161	5.008	0.026	0.010	0.608
R_temporalPOle	0.295	4.936	0.027	0.010	0.602

R_latfusif	0.206	4.839	0.028	0.010	0.593
L_stg	0.250	4.773	0.029	0.010	0.587
R_precentS	0.144	4.734	0.030	0.010	0.584
L_occ_m	0.115	4.711	0.030	0.010	0.582
L_mfg	0.092	4.649	0.032	0.010	0.576
L_plan_tempor_s	0.180	4.454	0.035	0.009	0.558
R_moccG	0.109	4.410	0.036	0.009	0.554
L_fiss_hor_ant	0.246	4.386	0.037	0.009	0.552
R_rectus	0.184	4.255	0.040	0.009	0.539
L_stg_lat	0.184	4.248	0.040	0.009	0.539
L_fis_ver_ant	0.238	4.202	0.041	0.009	0.534
R_paracentr	0.165	4.197	0.041	0.009	0.534
R_supTrans	0.095	4.103	0.043	0.009	0.525
L_octempSul_lat	0.151	4.089	0.044	0.009	0.523
R_orbG	0.109	3.964	0.047	0.008	0.511

without Sydney group

Region	Mean Square	F	Sig.	Partial Eta Squared	Observed Power
R_planumtempS	0.716	18.294	0.000	0.044	0.989
R_occAnt_sul	0.465	14.125	0.000	0.034	0.963
L_octemps_ML	0.362	14.105	0.000	0.034	0.963
L_collaTr_ants	0.551	13.097	0.000	0.032	0.951
R_supTempsul	0.221	12.349	0.000	0.030	0.939
R_precuneus	0.329	12.048	0.001	0.029	0.934
L_occAnt_sulc	0.417	11.163	0.001	0.027	0.915
R_occtemp_ML.s	0.302	10.989	0.001	0.027	0.911
R_ips	0.185	9.949	0.002	0.024	0.882
L_octempSul_lat	0.313	8.511	0.004	0.021	0.829
L_sup_par	0.229	8.419	0.004	0.021	0.825
R_parietalS	0.216	8.299	0.004	0.020	0.820
L_ifg_orb	0.363	8.249	0.004	0.020	0.817
R_insSHort	0.704	7.821	0.005	0.019	0.797
R_temporalPOle	0.454	7.585	0.006	0.019	0.785
L_temp_pole	0.426	7.572	0.006	0.019	0.784
R_ifgTr	0.225	7.438	0.007	0.018	0.777
R_cuneus	0.144	7.175	0.008	0.018	0.762
L_rectus	0.234	7.145	0.008	0.018	0.760
L_ITG	0.245	7.139	0.008	0.018	0.760
L_ifg_op	0.168	6.879	0.009	0.017	0.744
R_mtg	0.222	6.867	0.009	0.017	0.744
R_frontopolTrs	0.203	6.830	0.009	0.017	0.741
R_precentrInf	0.170	6.756	0.010	0.017	0.737
R_ifg_op	0.197	6.614	0.010	0.016	0.728
R_latFissureP	0.142	6.474	0.011	0.016	0.718

L_precuneus	0.178	6.376	0.012	0.016	0.712
R_itg	0.199	6.142	0.014	0.015	0.696
R_occsupG	0.173	6.012	0.015	0.015	0.687
R_transpost	0.237	5.955	0.015	0.015	0.682
R_infang	0.139	5.858	0.016	0.014	0.675
R_subcall	0.825	5.787	0.017	0.014	0.670
L_sfg	0.132	5.748	0.017	0.014	0.667
L_ips	0.097	5.676	0.018	0.014	0.662
L_occ_m	0.134	5.477	0.020	0.014	0.646
L_precentralg	0.294	5.373	0.021	0.013	0.638
R_mfg	0.102	5.326	0.022	0.013	0.634
L_fiss_hor_ant	0.301	5.311	0.022	0.013	0.633
L_stg_lat	0.224	5.284	0.022	0.013	0.631
R_cingMP	0.132	5.196	0.023	0.013	0.623
L_parietoOc	0.103	5.151	0.024	0.013	0.620
L_infTempSul	0.147	5.092	0.025	0.013	0.615
R_stglat	0.214	5.030	0.025	0.012	0.609
L_stg	0.255	4.988	0.026	0.012	0.606
L_inf_angular	0.127	4.874	0.028	0.012	0.596
L_cingMarg_s	0.118	4.840	0.028	0.012	0.593
R_latfusif	0.198	4.836	0.028	0.012	0.592
L_occ_s	0.150	4.705	0.031	0.012	0.581
R_moccG	0.114	4.522	0.034	0.011	0.564
L_plan_polsup	0.394	4.496	0.035	0.011	0.562
R_octemp_ML	0.075	4.398	0.037	0.011	0.553
L_fis_post	0.102	4.345	0.038	0.011	0.548
R_rectus	0.183	4.310	0.039	0.011	0.544
L_plan_tempor_s	0.173	4.309	0.039	0.011	0.544
L_central	0.071	4.178	0.042	0.010	0.532
L_cuneus	0.075	4.115	0.043	0.010	0.525
L_paracentral_g	0.165	4.113	0.043	0.010	0.525
L_ifg_triang	0.114	3.967	0.047	0.010	0.511
R_precentr	0.230	3.965	0.047	0.010	0.511
R_Jenses	0.171	3.947	0.048	0.010	0.509
L_inf_smg	0.102	3.932	0.048	0.010	0.507
R_octemp_Lat	0.146	3.912	0.049	0.010	0.505
R_sfg	0.080	3.798	0.052	0.009	0.494

without Oxford group

Region	Mean Square	F	Sig.	Partial Eta Squared	Power
L_octemps_ML	0.345	13.220	0.000	0.034	0.952
L_sup_par	0.350	13.144	0.000	0.034	0.951
R_planumtempS	0.453	11.607	0.001	0.030	0.925
R_occtemp_ML.s	0.296	10.944	0.001	0.028	0.910

R_precuneus	0.301	10.848	0.001	0.028	0.907
L_ips	0.184	10.561	0.001	0.027	0.900
R_ips	0.192	10.179	0.002	0.026	0.889
R_parietalS	0.240	9.157	0.003	0.024	0.855
L_ifg_op	0.228	9.150	0.003	0.024	0.855
L_precuneus	0.251	8.761	0.003	0.023	0.840
R_subcall	1.200	8.638	0.003	0.022	0.834
L_cingMarg_s	0.210	8.414	0.004	0.022	0.825
R_mfg	0.165	8.401	0.004	0.022	0.824
R_frontopolTrs	0.256	8.393	0.004	0.022	0.824
R_supTempsul	0.145	8.163	0.005	0.021	0.813
R_occAnt_sul	0.254	7.510	0.006	0.019	0.780
R_precentrInf	0.183	7.492	0.006	0.019	0.779
L_inf_angular	0.195	7.470	0.007	0.019	0.778
L_cuneus	0.139	7.400	0.007	0.019	0.774
R_insSHort	0.655	7.313	0.007	0.019	0.770
L_temp_pole	0.383	7.243	0.007	0.019	0.766
L_ifg_orb	0.326	7.134	0.008	0.019	0.759
L_occAnt_sulc	0.258	7.018	0.008	0.018	0.753
L_sfg	0.162	7.010	0.008	0.018	0.752
R_ifgTr	0.205	6.477	0.011	0.017	0.719
R_octemp_ML	0.112	6.326	0.012	0.016	0.708
R_ifg_op	0.192	6.270	0.013	0.016	0.705
R_itg	0.209	6.250	0.013	0.016	0.703
L_ITG	0.226	6.247	0.013	0.016	0.703
R_ifg_orb	0.321	5.542	0.019	0.014	0.651
L_stg_lat	0.247	5.518	0.019	0.014	0.649
L_stg	0.290	5.480	0.020	0.014	0.646
R_latfusif	0.229	5.455	0.020	0.014	0.644
R_cuneus	0.101	5.153	0.024	0.013	0.620
R_subpar	0.156	4.993	0.026	0.013	0.606
R_latFissureP	0.108	4.929	0.027	0.013	0.600
R_supFr	0.093	4.827	0.029	0.013	0.592
L_fis_ver_ant	0.263	4.781	0.029	0.012	0.587
R_orbG	0.128	4.756	0.030	0.012	0.585
L_precentralg	0.260	4.715	0.031	0.012	0.582
L_inf_smg	0.122	4.694	0.031	0.012	0.580
R_precentr	0.270	4.676	0.031	0.012	0.578
R_infang	0.110	4.622	0.032	0.012	0.573
R_mtg	0.154	4.611	0.032	0.012	0.572
L_ifs	0.081	4.592	0.033	0.012	0.570
L_ifg_triang	0.132	4.570	0.033	0.012	0.568
L_parietoOc	0.090	4.539	0.034	0.012	0.566
R_precentS	0.130	4.328	0.038	0.011	0.546
R_sfg	0.091	4.180	0.042	0.011	0.532

L_mfg	0.081	4.114	0.043	0.011	0.525
R_octemp_Lat	0.158	4.036	0.045	0.011	0.518
R_occsupG	0.115	3.999	0.046	0.010	0.514
L_occ_s	0.127	3.994	0.046	0.010	0.513
L_paracentral_g	0.161	3.966	0.047	0.010	0.511
L_fiss_hor_ant	0.224	3.943	0.048	0.010	0.508
R_latfissAntvert	0.324	3.872	0.050	0.010	0.501
R_temporalPOle	0.219	3.812	0.052	0.010	0.495
L_ins_g_short	0.284	3.800	0.052	0.010	0.494
L_ins_g	0.288	3.770	0.053	0.010	0.491
L_sfs	0.078	3.735	0.054	0.010	0.487

without Hong Kong group

Region	Mean Square	F	Sig.	Partial Eta Squared	Power
R_precuneus	0.506	17.760	0.000	0.037	0.988
L_sup_par	0.473	17.266	0.000	0.036	0.986
R_ips	0.322	17.132	0.000	0.036	0.985
L_ips	0.269	15.525	0.000	0.033	0.976
R_parietalS	0.400	15.319	0.000	0.032	0.974
R_planumtempS	0.581	14.960	0.000	0.031	0.971
L_octemps_ML	0.386	14.835	0.000	0.031	0.970
R_supTempsul	0.244	13.663	0.000	0.029	0.958
L_cingMarg_s	0.336	13.499	0.000	0.028	0.956
L_precuneus	0.357	12.417	0.000	0.026	0.940
R_mfg	0.236	12.383	0.000	0.026	0.940
L_occAnt_sulc	0.455	12.238	0.001	0.026	0.937
L_inf_angular	0.314	12.026	0.001	0.025	0.933
R_occAnt_sul	0.391	11.533	0.001	0.024	0.923
L_ITG	0.381	10.878	0.001	0.023	0.908
R_ifgTr	0.331	10.792	0.001	0.023	0.906
R_precentrInf	0.266	10.739	0.001	0.023	0.905
R_insSHort	0.950	10.582	0.001	0.022	0.901
L_sfg	0.242	10.517	0.001	0.022	0.899
R_cuneus	0.205	9.784	0.002	0.021	0.877
R_frontopolTrs	0.292	9.548	0.002	0.020	0.869
L_ifg_orb	0.424	9.487	0.002	0.020	0.867
L_ifg_op	0.234	9.472	0.002	0.020	0.867
L_temp_pole	0.522	9.434	0.002	0.020	0.865
L_parietoOc	0.183	9.117	0.003	0.019	0.854
R_occtemp_ML.s	0.244	8.838	0.003	0.019	0.843
R_occsupG	0.252	8.752	0.003	0.019	0.839
L_plan_tempor_s	0.352	8.650	0.003	0.018	0.835
R_supFr	0.160	8.461	0.004	0.018	0.827
L_cuneus	0.160	8.429	0.004	0.018	0.826

R_subpar	0.267	8.339	0.004	0.018	0.822
R_infang	0.197	8.228	0.004	0.018	0.817
R_latFissureP	0.174	8.100	0.005	0.017	0.811
R_mtg	0.272	8.090	0.005	0.017	0.810
R_precentS	0.236	7.846	0.005	0.017	0.798
R_sfg	0.164	7.737	0.006	0.017	0.793
L_occ_s	0.242	7.600	0.006	0.016	0.786
R_octemp_ML	0.131	7.404	0.007	0.016	0.775
L_inf_smg	0.190	7.297	0.007	0.016	0.769
R_precentr	0.410	7.128	0.008	0.015	0.759
L_ifs	0.120	6.917	0.009	0.015	0.747
L_mfg	0.133	6.742	0.010	0.014	0.736
R_itg	0.223	6.741	0.010	0.014	0.736
R_ifg_op	0.201	6.683	0.010	0.014	0.732
R_subcall	0.919	6.629	0.010	0.014	0.729
R_moccG	0.162	6.566	0.011	0.014	0.725
L_fiss_hor_ant	0.369	6.554	0.011	0.014	0.724
L_stg_lat	0.284	6.552	0.011	0.014	0.724
R_temporalPOle	0.388	6.516	0.011	0.014	0.722
L_paracentral_g	0.260	6.475	0.011	0.014	0.719
L_trsv	0.141	6.212	0.013	0.013	0.701
R_supTrans	0.145	6.209	0.013	0.013	0.701
R_latfusif	0.260	6.132	0.014	0.013	0.695
L_precentralg	0.320	5.814	0.016	0.012	0.672
L_collaTr_ants	0.251	5.703	0.017	0.012	0.664
R_ifg_orb	0.317	5.663	0.018	0.012	0.661
L_ifg_triang	0.163	5.624	0.018	0.012	0.658
R_paracentr	0.210	5.411	0.020	0.012	0.641
L_central	0.092	5.375	0.021	0.012	0.638
R_orbG	0.146	5.306	0.022	0.011	0.633
R_inffrontsulc	0.090	5.090	0.025	0.011	0.615
R_octemp_Lat	0.198	5.033	0.025	0.011	0.610
R_Jenses	0.213	5.020	0.026	0.011	0.609
L_stg	0.263	4.986	0.026	0.011	0.606
L_occ_m	0.123	4.960	0.026	0.011	0.604
R_INfparsupramarg	0.135	4.928	0.027	0.011	0.601
L_rectus	0.165	4.824	0.029	0.010	0.592
R_parietoOcc	0.087	4.802	0.029	0.010	0.590
R_stglat	0.204	4.687	0.031	0.010	0.579
L_sfs	0.095	4.617	0.032	0.010	0.573
L_ins_g_short	0.327	4.441	0.036	0.010	0.557
R_cingMP	0.109	4.245	0.040	0.009	0.538
L_fis_post	0.099	4.190	0.041	0.009	0.533
L_octempSul_lat	0.155	4.187	0.041	0.009	0.533
R_rectus	0.179	4.150	0.042	0.009	0.529

R_latfissAntvert	0.325	3.890	0.049	0.008	0.503
L_fusi	0.132	3.851	0.050	0.008	0.499

without UCL group

Region	Mean Square	F	Sig.	Partial Eta Squared	Power
L_octemps_ML	0.442	17.300	0.000	0.043	0.986
R_precuneus	0.425	15.325	0.000	0.038	0.974
R_ifgTr	0.465	15.014	0.000	0.038	0.972
L_sup_par	0.384	14.193	0.000	0.036	0.964
R_planumtempS	0.542	13.550	0.000	0.034	0.957
R_supTempsul	0.239	13.236	0.000	0.033	0.952
R_parietalS	0.313	12.155	0.001	0.031	0.935
L_cingMarg_s	0.297	12.062	0.001	0.030	0.934
R_frontopolTrs	0.333	11.201	0.001	0.028	0.916
R_subcall	1.483	11.169	0.001	0.028	0.915
L_precuneus	0.314	11.080	0.001	0.028	0.913
L_ips	0.191	11.052	0.001	0.028	0.912
L_ifg_orb	0.487	10.985	0.001	0.028	0.911
R_mfg	0.209	10.818	0.001	0.027	0.907
L_sfg	0.237	10.310	0.001	0.026	0.893
R_insSHort	0.892	10.226	0.001	0.026	0.891
R_ips	0.192	10.095	0.002	0.026	0.887
L_temp_pole	0.543	10.086	0.002	0.026	0.886
R_occAnt_sul	0.335	10.041	0.002	0.025	0.885
R_occtemp_ML.s	0.266	9.747	0.002	0.025	0.876
L_parietoOc	0.193	9.610	0.002	0.024	0.871
L_ITG	0.318	9.603	0.002	0.024	0.871
L_cuneus	0.168	9.302	0.002	0.024	0.860
L_rectus	0.277	8.413	0.004	0.021	0.825
L_inf_angular	0.215	8.283	0.004	0.021	0.819
L_ifs	0.146	8.278	0.004	0.021	0.819
L_occAnt_sulc	0.303	8.199	0.004	0.021	0.815
R_precentrInf	0.206	8.178	0.004	0.021	0.814
R_latFissureP	0.177	8.021	0.005	0.020	0.806
R_cuneus	0.157	7.790	0.006	0.020	0.795
R_supFr	0.147	7.760	0.006	0.020	0.794
L_ifg_triang	0.226	7.751	0.006	0.020	0.793
R_ifg_op	0.226	7.414	0.007	0.019	0.775
R_inffrontsulc	0.129	7.222	0.008	0.018	0.764
L_inf_smg	0.188	7.156	0.008	0.018	0.761
R_mtg	0.226	6.757	0.010	0.017	0.737
R_moccG	0.164	6.620	0.010	0.017	0.728
R_orbG	0.173	6.559	0.011	0.017	0.724
R_ifg_orb	0.372	6.556	0.011	0.017	0.724

R_octemp_ML	0.104	6.194	0.013	0.016	0.699
L_ifg_op	0.148	5.977	0.015	0.015	0.684
L_occ_s	0.188	5.864	0.016	0.015	0.676
R_itg	0.189	5.771	0.017	0.015	0.669
R_subpar	0.186	5.736	0.017	0.015	0.666
R_INfparsupramarg	0.159	5.719	0.017	0.015	0.665
L_insulaA	0.262	5.424	0.020	0.014	0.642
L_sfs	0.113	5.415	0.020	0.014	0.641
L_fis_ver_ant	0.304	5.406	0.021	0.014	0.640
R_supTrans	0.127	5.388	0.021	0.014	0.639
R_Jenses	0.233	5.365	0.021	0.014	0.637
L_fis_post	0.123	5.272	0.022	0.014	0.629
L_paracentral_g	0.208	5.175	0.023	0.013	0.621
L_octempSul_lat	0.195	5.169	0.024	0.013	0.621
L_stg	0.273	5.143	0.024	0.013	0.619
L_occ_m	0.124	5.122	0.024	0.013	0.617
R_infang	0.122	5.096	0.025	0.013	0.615
L_precentrInf	0.135	5.047	0.025	0.013	0.611
R_parietoOcc	0.090	5.024	0.026	0.013	0.609
R_temporalPOle	0.301	5.003	0.026	0.013	0.607
R_octemp_Lat	0.187	4.852	0.028	0.012	0.594
L_plan_tempor_s	0.189	4.642	0.032	0.012	0.575
R_occsupG	0.130	4.450	0.036	0.011	0.557
R_sfg	0.094	4.424	0.036	0.011	0.555
L_infTempSul	0.123	4.303	0.039	0.011	0.544
L_mfg	0.086	4.288	0.039	0.011	0.542
R_transpost	0.173	4.202	0.041	0.011	0.534
R_precentr	0.241	4.165	0.042	0.011	0.530
R_latfissAntvert	0.334	3.967	0.047	0.010	0.511
L_fiss_hor_ant	0.222	3.958	0.047	0.010	0.510
R_rectus	0.161	3.815	0.052	0.010	0.495
L_ins_g	0.277	3.743	0.054	0.010	0.488

Surface area ANCOVAs (age, gender and TIV as covariates)

Results are fully consistent with those from the main sample.

without Lille group

Region	*mean square*	*df*	*F*	*partial eta2*	*p*	*obs. power*
R_occtemp ML	524443,587	1,461	7,220	0,016	0,007	0,765

without Yonsei group

Region	*mean square*	*df*	*F*	*partial eta2*	*p*	*obs. power*
R_occtemp ML	784851,915	1,395	9,898	0,025	0,002	0,881

without Newcastle group

Region	mean square	df	F	partial eta2	p	obs. power
R_occtemp ML	629800,775	1,472	8,665	0,018	0,003	0,836

without KCL group

Region	mean square	df	F	partial eta2	p	obs. power
R_occtemp ML	831137,731	1,476	11,346	0,023	0,001	0,919

without Sydney group

Region	mean square	df	F	partial eta2	p	obs. power
R_occtemp ML	771329,198	1,404	10,685	0,026	0,001	0,903

without Oxford group

Region	mean square	df	F	partial eta2	p	obs. power
R_occtemp ML	830055,090	1,382	11,872	0,03	0,001	0,930

without Hong Kong group

Region	mean square	df	F	partial eta2	p	obs. power
R_occtemp ML	590105,105	1,465	8,089	0,017	0,005	0,810

without UCL group

Region	mean square	df	F	partial eta2	p	obs. power
R_occtemp ML	613551,331	1,389	8,418	0,021	0,004	0,825

Volume MANCOVAs (age, gender and TIV as covariates)

The leave-one-out analysis showed that the volume differences were dependent on the inclusion of the Sydney group. While the small MMSE difference was significant in this group, this was one of the two groups with the largest VH group, so it is difficult to understand if the differences in volume results in the leave-one-out analysis were due to the variation in statistical power. However, given that in the N=146 subsample (NPI) the Sydney group is not included and we found the same difference in the amygdala plus the hippocampus when adding the relevant available covariates, the contribution of these regions to PH-VH is supported by our data overall.

without Lille group

Region	mean square	df	F	partial eta2	p	obs. power
Left-Cerebellum-White-Matter	21148259,518	1,457	3,993	0,009	0,046	0,514
Left-Amygdala	198677,703	1,457	3,760	0,008	0,053	0,490
Right-Amygdala	223700,951	1,457	4,327	0,009	0,038	0,546

without Yonsei group

Region	mean square	df	F	partial eta2	p	obs. power
Left-Cerebellum-White-Matter	29759925,583	1,395	5,213	0,013	0,023	0,625
Left-Amygdala	213275,446	1,395	3,783	0,010	0,053	0,492
Right-Amygdala	286612,852	1,395	5,033	0,013	0,025	0,609

without Newcastle group

Region	*mean square*	*df*	*F*	*partial eta2*	*p*	*obs. power*
Left-Cerebellum-White-Matter	18300727,667	1,472	3,573	0,008	0,059	0,471
Left-Amygdala	289031,643	1,472	5,503	0,012	0,019	0,648
Right-Amygdala	211263,858	1,472	4,050	0,009	0,045	0,519

without KCL group

Region	*mean square*	*df*	*F*	*partial eta2*	*p*	*obs. power*
Left-Cerebellum-White-Matter	20441807,659	1,476	3,843	0,008	0,051	0,499
Left-Amygdala	375712,039	1,476	7,272	0,015	0,007	0,768
Right-Amygdala	297646,868	1,476	5,853	0,012	0,016	0,675

without Sydney group

Region	*mean square*	*df*	*F*	*partial eta2*	*p*	*obs. power*
Left-Cerebellum-White-Matter	11151129,788	1,404	2,189	0,005	0,140	0,314
Left-Amygdala	154059,211	1,404	2,900	0,007	0,089	0,397
Right-Amygdala	50705,368	1,404	0,944	0,002	0,332	0,163

without Oxford group

Region	*mean square*	*df*	*F*	*partial eta2*	*p*	*obs. power*
Left-Cerebellum-White-Matter	17566193,828	1,382	3,559	0,009	0,060	0,469
Left-Amygdala	462292,677	1,382	9,160	0,024	0,003	0,855
Right-Amygdala	356095,410	1,382	7,174	0,019	0,008	0,762

without Hong Kong group

Region	*mean square*	*df*	*F*	*partial eta2*	*p*	*obs. power*
Left-Cerebellum-White-Matter	27485445,952	1,465	5,255	0,011	0,022	0,628
Left-Amygdala	431005,217	1,465	8,209	0,017	0,004	0,816
Right-Amygdala	330712,956	1,465	6,447	0,014	0,011	0,717

without UCL group

Region	*mean square*	*df*	*F*	*partial eta2*	*p*	*obs. power*
Left-Cerebel-WM	33881207,505	1,389	6,654	0,017	0,010	0,730
Left-Amygdala	376353,666	1,389	7,686	0,020	0,006	0,790
Right-Amygdala	268675,762	1,389	5,386	0,014	0,021	0,639

Supplementary Note 4. *NPI subgroup custom MANCOVAs with binary score (PD-VH vs. PD-noVH) and correlation analysis (VH only) with LED as covariate.*

The NPI subgroup (146 patients, 79 PD-noVH, 67 PD-VH) is the group for which we had the richest clinical dataset, as for most of these patients we have not only age and gender, but also disease onset, levo-dopa equivalent daily dose (LED) and mini mental state examination (MMSE) data, PD severity, together with the continuous NPI score. 126 had UPDRS-III scores. 20 from the same group did not have those scores. We computed the mean for the group regardless of VH and used that score in our model.

Patients do not differ gender (35 females PD-noVH, 27 females PD-VH) ($\chi 2$ = .238, p = 0.62), age (PD-VH=70.39 ± 6.82, PD-noVH = 69.64 ± 6.45) [$F(87,47)$ = 0.47, *MSE* = 20.39, p = .5], disease onset (PD-VH=5.86 ± 5.40, PD-noVH = 4.64 ± 5.22) [$F(87,47)$ = 1.91, *MSE* = 53.85, p = .17], levodopa equivalent daily dose (LED) (PD-VH=575.86 ± 366.43 mg, PD-noVH =502.17± 327.34 mg) [$F(87,47)$ =1.60, *MSE* =191615.74, p = .21], and MMSE score (PD-VH=26.28±2.69, PD-noVH =26.81±2.51), [$F(87,47)$=1.49, *MSE* = 10.05, p = .22)].

Patients were matched for UPDRS-III within each study. However, when carrying out a one-way ANOVA using the mean to fill the 20 missing values (N PD-VH =7 , N PD-noVH = 13) they were not matched for UPDRS-III, with PD-VH having a higher score [$F(1,113)$=6.9, *MSE* = 1187.07, p = .01)].

We ran a custom multivariate ANOVA model with MMSE, gender, age, TIV, UPDRS-III, LED and onset as covariates, controlling also for the interaction of those covariates that correlated with each other (age * LED + age * MMSE + onset * LED + onset * UPDRS3 +LED * MMSE).

Supplementary Table 4(a). Demographics

Group	*Age*	*MMSE*	*LED*	*Onset*	*UPDRS-III*
PD-VH (N=67, 27 F)	70.39 (SD=6.82)	26.28 (SD =2.69)	575.86 mg (SD =366.43)	5.86 (SD =5.40)	30.32 (SD =15.25)
	p = .49	p = .22	p = .21	p = .17	$\mathbf{p = .01}$
PD-noVH (N=79, 35 F)	69.64 (SD =6.45)	26.81(SD =2.51)	503.17 mg (SD =327.34)	4.64 (SD =5.22)	24.61 (SD =10.93)

Supplementary Table 4(b). *The table below report results of the between-subjects effects tests on the different regions when comparing PD-VH and PD-noVH as described in Supplementary note 4. Pairwise comparisons are Bonferroni corrected. First, we present a summary of the demographics and clinical information of this subsample; for UPDRS-III values, we report the scores including the N=20 subjects with missing values; for original UPDRS-III scores for each group see* ***Table 1*** *in the main text (study: Yonsei University; University of Newcastle; University of Lille)*

Cortical thickness

**Region**	**df**	**MSE**	**F**	**p**	**partial eta2**	**power**
R_planumtemp	1.133	0.57	16.22	0.00	0.11	0.98

L_cing.marg	1.133	0.28	13.28	0.00	0.09	0.95
R_sts	1.133	0.22	13.02	0.00	0.09	0.95
L_occtemp.ml.sul	1.133	0.23	10.31	0.00	0.07	0.89
R_smg	1.133	0.26	9.80	0.00	0.07	0.87
R_jensen(ips)	1.133	0.39	9.71	0.00	0.07	0.87
L_coll.trsv	1.133	0.39	9.50	0.00	0.07	0.86
R_ips	1.133	0.16	9.39	0.00	0.07	0.86
L_sup.parg	1.133	0.24	8.58	0.00	0.06	0.83
R_occtemp.ml.sul	1.133	0.20	8.43	0.00	0.06	0.82
L_temp.pole	1.133	0.41	8.41	0.00	0.06	0.82
L_occtem.lat.sul	1.133	0.27	7.13	0.01	0.05	0.76
R_parietalS	1.133	0.18	7.11	0.01	0.05	0.75
L_ifs	1.133	0.12	7.08	0.01	0.05	0.75
R_mtg	1.133	0.22	7.03	0.01	0.05	0.75
R_precent.inf.sul	1.133	0.17	6.80	0.01	0.05	0.74
L_ips	1.133	0.12	6.78	0.01	0.05	0.73
R_occ.m.g	1.133	0.16	6.49	0.01	0.05	0.72
R_occ.ant.sulc	1.133	0.20	6.47	0.01	0.05	0.71
R_precuneus	1.133	0.16	6.33	0.01	0.05	0.70
R_latfis	1.133	0.14	6.28	0.01	0.05	0.70
R_transv.post	1.133	0.24	6.07	0.02	0.04	0.69
R_cuneus	1.133	0.12	5.91	0.02	0.04	0.68
L_fiss.hor	1.133	0.29	5.71	0.02	0.04	0.66
R_ifg_orb	1.133	0.36	5.64	0.02	0.04	0.65
R_ifg_op	1.133	0.17	5.61	0.02	0.04	0.65
R_itg	1.133	0.17	5.58	0.02	0.04	0.65
L_paracentral g	1.133	0.23	5.58	0.02	0.04	0.65
R_ifg_tri	1.133	0.18	5.54	0.02	0.04	0.65
L_precuneus	1.133	0.15	5.54	0.02	0.04	0.65
L_itg	1.133	0.18	5.44	0.02	0.04	0.64
R_ifs	1.133	0.10	5.43	0.02	0.04	0.64
L_occ.ant.sul	1.133	0.19	5.33	0.02	0.04	0.63
R_parietocc.sul	1.133	0.08	5.23	0.02	0.04	0.62
R_angular	1.133	0.11	5.23	0.02	0.04	0.62
L_planum.pol	1.133	0.44	5.21	0.02	0.04	0.62
R_occtemp.lat.s	1.133	0.20	5.06	0.03	0.04	0.61
L_its	1.133	0.13	5.01	0.03	0.04	0.60
R_mfg	1.133	0.09	4.58	0.03	0.03	0.57
L_precentral.g	1.133	0.24	4.47	0.04	0.03	0.56
R_frontopolar.gs	1.133	0.12	4.37	0.04	0.03	0.55
L_occ.m g	1.133	0.10	4.29	0.04	0.03	0.54
R_subpar.s	1.133	0.11	4.23	0.04	0.03	0.53
L_ifg_tri	1.133	0.11	4.09	0.05	0.03	0.52
L_parietocc	1.133	0.09	3.92	0.05	0.03	0.50
L_ifg_op	1.133	0.16	3.83	0.05	0.03	0.49

Subcortical volumes

Region	*df*	*ms*	*F*	*p*	*partial eta2*	*obs. Power*
L.Hippocampus	1,146	702601.17	6.528	0.012	0.047	0.718
L.Amygdala	1,146	221211.382	7.011	0.009	0.050	0.748
R.Caudate	1,146	844168.4132	4.691	0.032	0.034	0.574
R.Hippocampus	1,146	582591.8887	3.745	0.05	0.027	0.483

Cortical thickness. *Multivariate analysis results for cortical thickness differences in PD+VH vs. PD noVH patients.* ***Subcortical volumes.*** *Multivariate analysis results for subcortical volume differences in PD+VH vs. PD noVH patients. Regions are sorted by p value. * values greater for PD with VH;*

For thickness, no significant main effect of age, MMSE, LED, UPDRS, onset, age * LED + age * MMSE + onset * LED + onset * UPDRS-III + LED * MMSE was observed. We found a significant main effect of gender [$F(87,47)=1.93$, $p=.008$] and TIV [$F(87,47)=2.28$, $p =.001$].

For subcortical volumes, we observed a main effect of gender [$F(17,117)=2.97$, $p<.001$] and TIV [$F(17,117)=28.73$, $p <.001$]. No significant main effect of age, MMSE, LED, UPDRS, onset, age * LED + age * MMSE + onset * LED + onset * UPDRS-III + LED * MMSE was observed.

Finally, we carried out the same correlational analysis described in the main text, but with LEDD as a covariate, as it might be related to severity of VH, obtaining the same results: negative correlations were found for the right superior temporal sulcus (r = -.26, p = .03), the right inferior parietal sulcus (r = -.24, p = .05), the right Jensen sulcus (r = -.28, p = .02) and the right cingulum marginalis (r = -.24, p = .056), which was a trend in this case. In addition there was the positive correlation reported also in the main text with the right frontomarginal gyrus (r = .26, p = .04).

Supplementary Note 5. *Subgroup analysis with ordinal VH score.*

An additional sensitivity analysis was carried out for a large subgroup, that is all those patients for which we had additional information about the degree of hallucinations, levodopa equivalent dose (LED) and onset date. Overall, the 128 PD-VH patients (64 mild, 47 moderate, 17 severe) were matched for age [F(2,125)=1.59, p=0.21], onset [F(2,125)=0.18, p=0.84], gender (X^2=.87, p=.65) and TIV [F(2,125)=0.07, p=0.93] (we did not have enough LED or cognition data to check for these variables).

Patients with mild VH had a mean age of 66.71± 8.20, those with moderate VH 69.10 ± 7.37 and those with severe VH 69.34 ± 7.30. Those with mild VH had a disease duration of 5.29± 4.06 years, those with moderate VH 5.57± 5.58 and those with severe 4.79 ± 4.05. Finally, patients with mild VH had a mean TIV of 1477284.73 ±178401.32, patients with moderate VH 1490485.07 ±188476.12, patients with severe VH 1480473.38 ±195998.02.

As there is not a specific criterion to do so, in order to create an ordinal variable, for patients we had UPDRS scoring we have retained that (0 = no hallucinations or delusions, 1= illusions or non formed hallucinations, 2 = Formed hallucinations independent of environmental stimuli, 3= Formed hallucinations with loss of insight, 4= patient has delusions or paranoia (but no patients had a UPDRS 4 score or a NPI, NEVHI, MIAMI equivalent). We have "translated" the NPI continuous score to such a scale, with scores ranging from 1 to 3 categorised as "minor/mild VH", scores raging from 4 to 8 as "moderate" and scores above 9 as "severe"; for the MIAMI we have used a similar procedure, as the scale ranges from 0 to 14. Finally, for the NEVHI, we have used the raw data from the interviews to determine whether the person had mild, moderate or severe visual hallucinations.

To isolate regions where there was some difference between the groups in order to create a multi-region ordinal regression model, we ran a multivariate ANCOVA for each morphometric measure (subcortical volumes, SA, cortical thickness) was ran with *degree of hallucinations as between subjects factor* on hallucinators only (McCrum-Gardner, 2008) and with age, onset, gender, TIV as covariates.

For thickness we used age [F(2,121)=1.25, p=0.62], gender[F(2,121)=1.95, p=0.52], onset [F(2,121)=.3.91, p=0.37], and TIV[F(2,121)=.455, p=.86], as covariates, finding no main effect for any of these factors except for TIV. Participants differed in thickness in the left temporal pole, left insula, left collateralis transversalis posterior, left suborbital gyrus, right frontomarginal gyrus (as in the NPI correlational analysis) and in the right lateral horizontal fissure, with severe and moderate hallucinators having higher thickness in these regions. We speculate that greater thickness in these regions may constitute a pre-existing risk factor for aggravation of VH severity but additional data would be needed to further explore this hypothesis.

For SA the covariates age [F(2,121)=.42, p=0.87], gender[F(2,121)=5.25, p=0.34], onset [F(2,121)=.88, p=0.68], TIV [F(2,121)=4.64, p=0.36] showed no main effect. Participants differed in SA in the occipitotemporal gyri and in the precuneus bilaterally, calcarine fissure, insula and precentral gyrus; SA was reduced in severe hallucinators if compared to mild and moderate hallucinators.

For subcortical volumes we found a main effect of age [F(2,121)=2.91, p<.001], gender[F(2,121)=2.02, p=0.003], and TIV[F(2,121)=769.44, p<.001], but not of onset [F(2,121)=1.37, p=0.11]. Participants differed in volume

in the bilateral inferior lateral ventricles, with moderate hallucinators having greater ventricle volume than mild hallucinators.

We entered the regions emerged as significant in ordinal logistic regression models carried out with R package MASS. For *thickness*, the intercepts for mild vs. moderate hallucinators (t =1.87, *st.error* = 3.41, *intercept* = 6.37, p=.06) showed a trend towards significant and was significant for moderate vs. severe (t =2.42, *st.error* = 3.45, *intercept* = 8.36, p =.01), were significant with *residual deviance* = 241.43, *AIC* = 257.43. Interestingly, thickness in these regions is greater in the moderate and severe hallucinators overall, recalling how the right frontomarginal thickness positively correlated with the NPI score in the smaller sample. However, none of the regions significantly predicted the hallucinations ordinal score (all t values < ±1.5 and all *ps*>.05).

For *surface area* mild vs. moderate hallucinators (t = -1529.65, *st.error* = 0.001, *intercept* = -1.71, p <.0001) was significant, but not for moderate vs. severe (t =1.44, *st.error* = 0.28, *intercept* = 0.41, p =.15), were significant with *residual deviance* = 231.47, *AIC* = 253.47, with the coefficients for the left occipitotemporal fusiform gyrus being above the ~2 threshold for the t value (t =-2.33, p =.001) and being greater in mild vs. moderate, and the left insula circularis showing the opposite pattern (t =2.87, p =.004). The lack of significance when considering the comparison with those with a 'severe' score may be due to different reasons, with one being that the different PDP scales used in the different studies have slightly different criteria, but also that patients with higher scores may have delusions as well, which may as well be in the same continuum as visual hallucinations, but this does not necessarily imply a graver atrophy in the same regions underlying VH. Finally, the three groups have very different sample sizes, and this might as well affect the analyses, as one can notice the large variability within each group.

Supplementary Note 6. *Additional models and details about receptor density maps.*

a) *Additional models for thickness.*

For regions where there was a relationship between receptor density and structural morphometrics for all regions, additional analyses were conducted to assess whether this was simply the same effect as the relationship of the regions that differed or a feature of the whole brain by examining the non-significant regions.

Cortical Thickness. The models with 5-HT2$_A$ binding potential per region as predictor and the mean difference per region (considering only the regions where no difference between groups was observed) as dependent variable resulted not significant for regions that did not differ (β = .04 , t= .32 p=0.75); a similar result was obtained for 5-HT1$_A$ (β =.03 , t=.28, p= 0.78). However the result was significant for D2/D3 (β =-.23 , t=-2.06.72, p= .04) for the same regions, possibly suggesting that D2/D3 alterations' relationship with thickness decrease go beyond the associating with VH in PD.

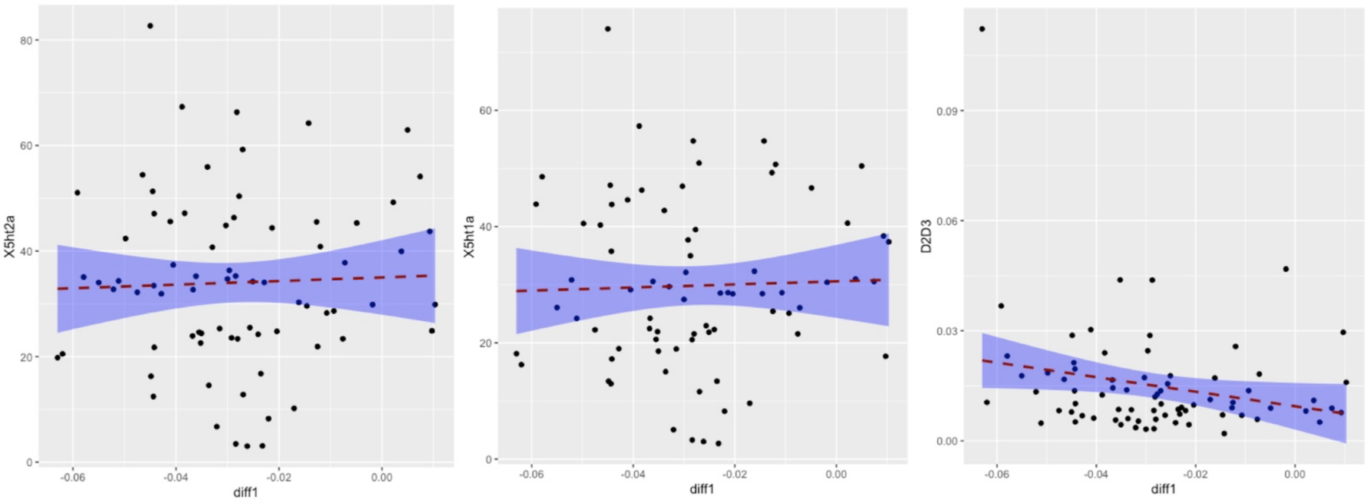

Supplementary Figure 3. Scatter plots for receptors density profiles and regions not differing in the two groups. Non significant results were found for serotonergic receptors, whereas the dopaminergic receptor resulted a significant predictor also in this model (as in the others presented in the main text). The shaded area represents the confidence interval.

b) *Receptors binding potentials models for volumes models results*.

Regression models were carried out to estimate the relationship between subcortical volumes (N=19) mean differences between hallucinators and non-hallucinators and receptor density profiles. As significantly differing regions were just the hippocampi and the amygdala, we ran separate models for each receptor using all regions' subcortical volumes. The models resulted not significant when 5-HT2$_A$ binding potential per region was used as predictor (β = -.14, t = -0.6, p = .5) , when 5-HT1$_A$ (β = -.24, t = -1.01, p = .32) and D2/D3 (β = .06, t = .24 , p = .82) were used as predictors.

c) In order to better understand these results, we ran Pearson's correlations between each receptor's density maps at the cortical level, finding that 5-HT2A and D2/D3 did now show a significant correlation (r =.15 p = .07), whereas 5-HT2A and 5-HT1A did (r =.92, p <.001) and also 5-HT1A and D2/D3 (r =.27, p =.002).

Supplementary Note 7. *Principal Component Analysis for cortical thickness: additional information about the scree plot and the contribution of the different regions to Dimension1 and Dimension 2.*

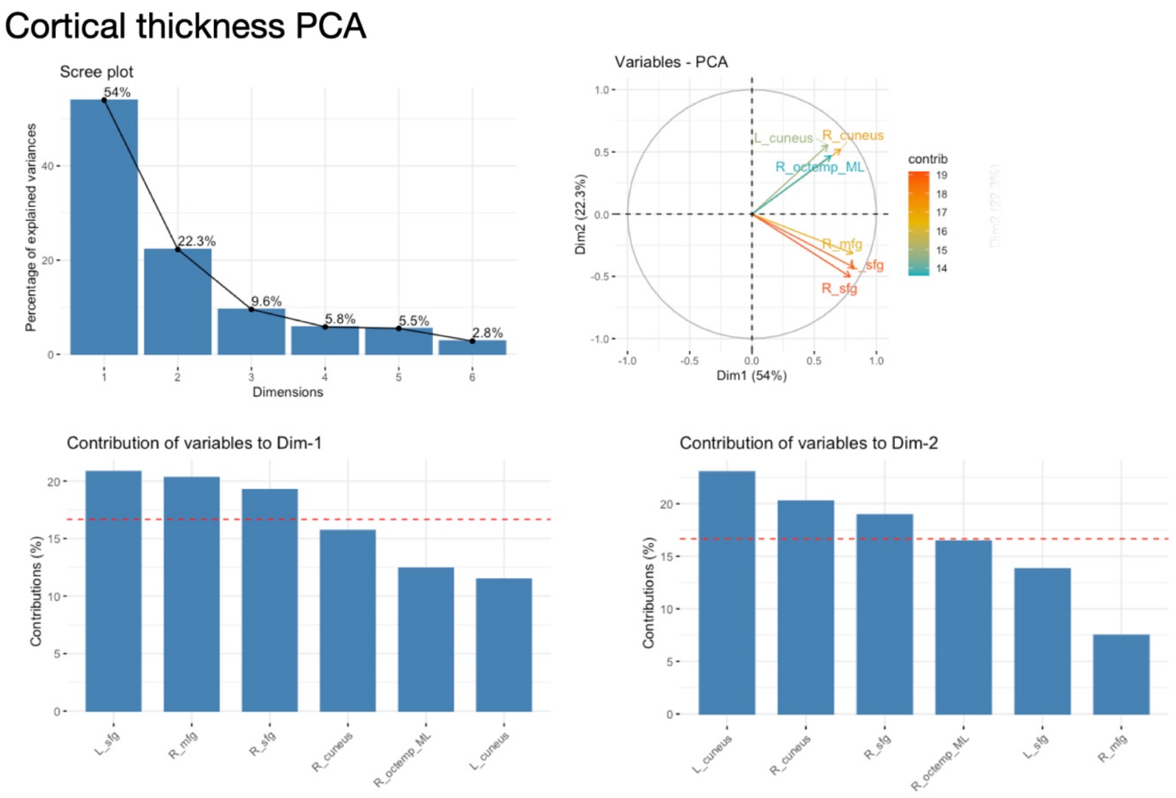

Supplementary Figure 4. ***Top left:*** *Scree Plot for Cortical thickness data. On the y axis, the % of variance explained by each component, on the y axis the components number. Following the criterion of picking only components with eigenvalues >1, PC 1 and 2 were selected.* ***Top right:*** *visual representation of the components and the dimensions. The colours represent the contributing weights and cosine squared.* ***Bottom row:*** *Contributing weight by region for each selected dimension.*

Supplementary Note 8. *Structural covariance.*

a) Differences in inter-regional correlations between PD-VH and PD-noVH represented with a correlogram. Covariance matrices computed on the GLM residuals used for the structural covariance analysis were used to compute the difference in the inter-regional correlation coefficients in [PD-VH – PDnoVH] patients (**Supplementary Figure 5**; those depicted are the difference in the coefficients). A cell by cell comparison of the correlation coefficients with the *cocor* package for R was computed to investigate regions that showed the highest covariance in PD-VH and in PD-noVH. In purple, inter-regional correlation coefficients that were greater for PD-VH, in yellow those greater for PD-noVH. The analysis on the z scores (Fischer) highlighted that the most significant regions with the most inter-regional correlation with other regions for VH patients were the left caudal MFG, left cuneus, left IPL, left ITG, left iCC, left LOG, left middle orbitofrontal gyrus, left paracentral gyrus, left IFG opercularis, left temporoparietal, right parahippocampal gyrus, right iFG opercularis, right IFG triangular, right temporoparietal. In particular the left cMFG highly correlated with parieto-occipital regions (IPL, cuneus. Lateral occipital gyrus, SPL, STG) and frontal (SFG), on both hemispheres; the left LOG with left SFG, MFG, STG, SMAR, and right IFG (all subdivision); the right PHG with temporal (left TP and STG), parietal (left precuneus, precentral) and occipitotemporal (right enthorinal, fusiform, inferior temporal) regions, mainly.

For PD-noVH patients, we found an overall greater significant of inter-regional correlation coefficients in the left and right pericallosal area, in the right lingual gyrus, and in the right superior frontal gyrus.

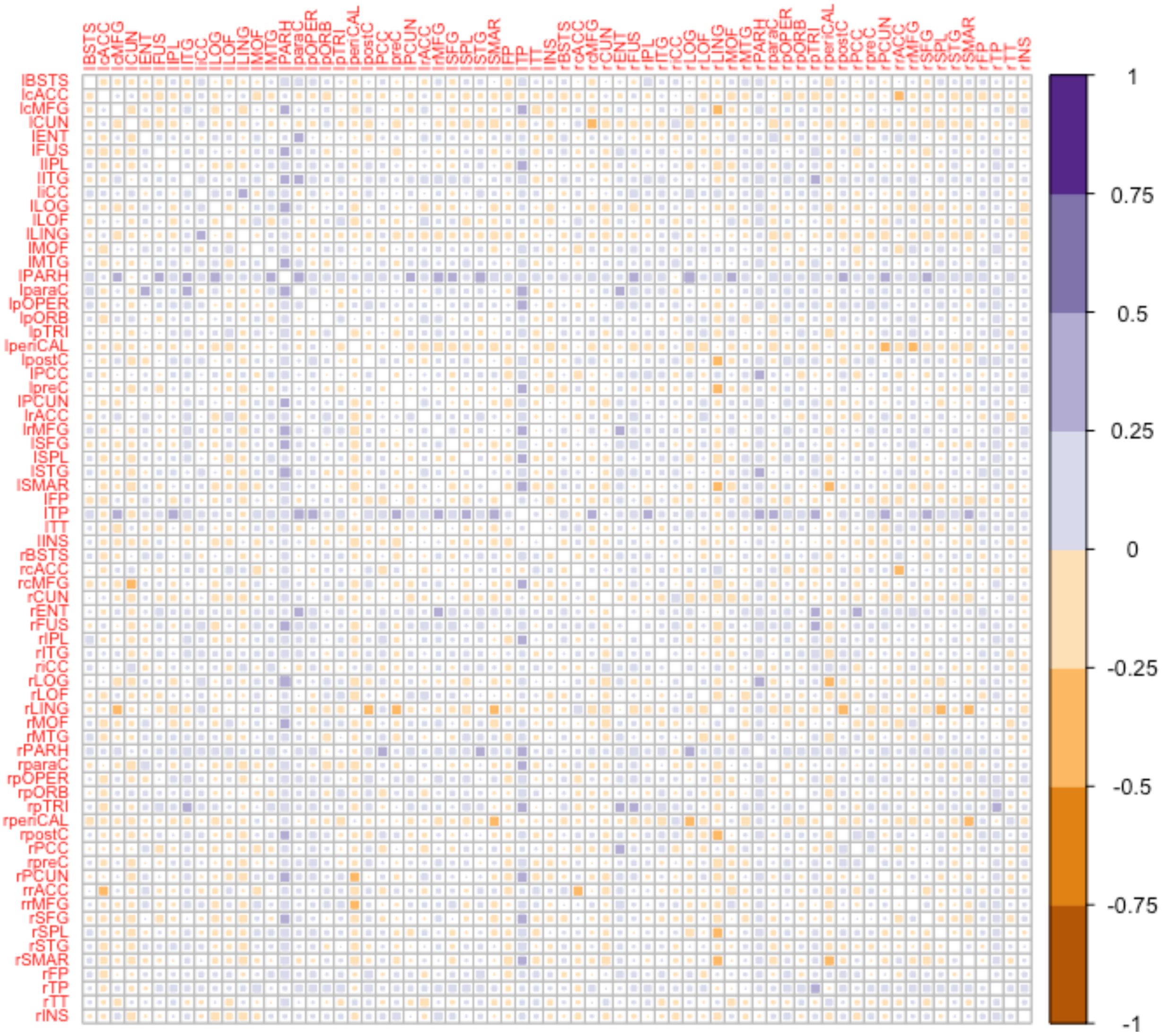

Supplementary Figure 5. *Correlogram showing the difference in inter-regional correlation coefficients in PD–VH vs. PD-noVH patients for cortical thickness. The yellow squares indicate inter-regional coefficients that were greater for PD-noVH; of those, only some were significant, mainly those in the left and right pericallosal column, in the right lingual column.*

The same procedure was carried out for *surface area*. However, we did not find any region where the correlation coefficient was greater for PD-noVH than for PD-VH. As for cortical thickness, regions with significant regions with the most inter-regional correlation with other regions were the left caudal MFG, left cuneus , left fusiform, right temporal transverse, right IFG triangualris, bilateral IFG opercularis and insula, IPL, SFG.

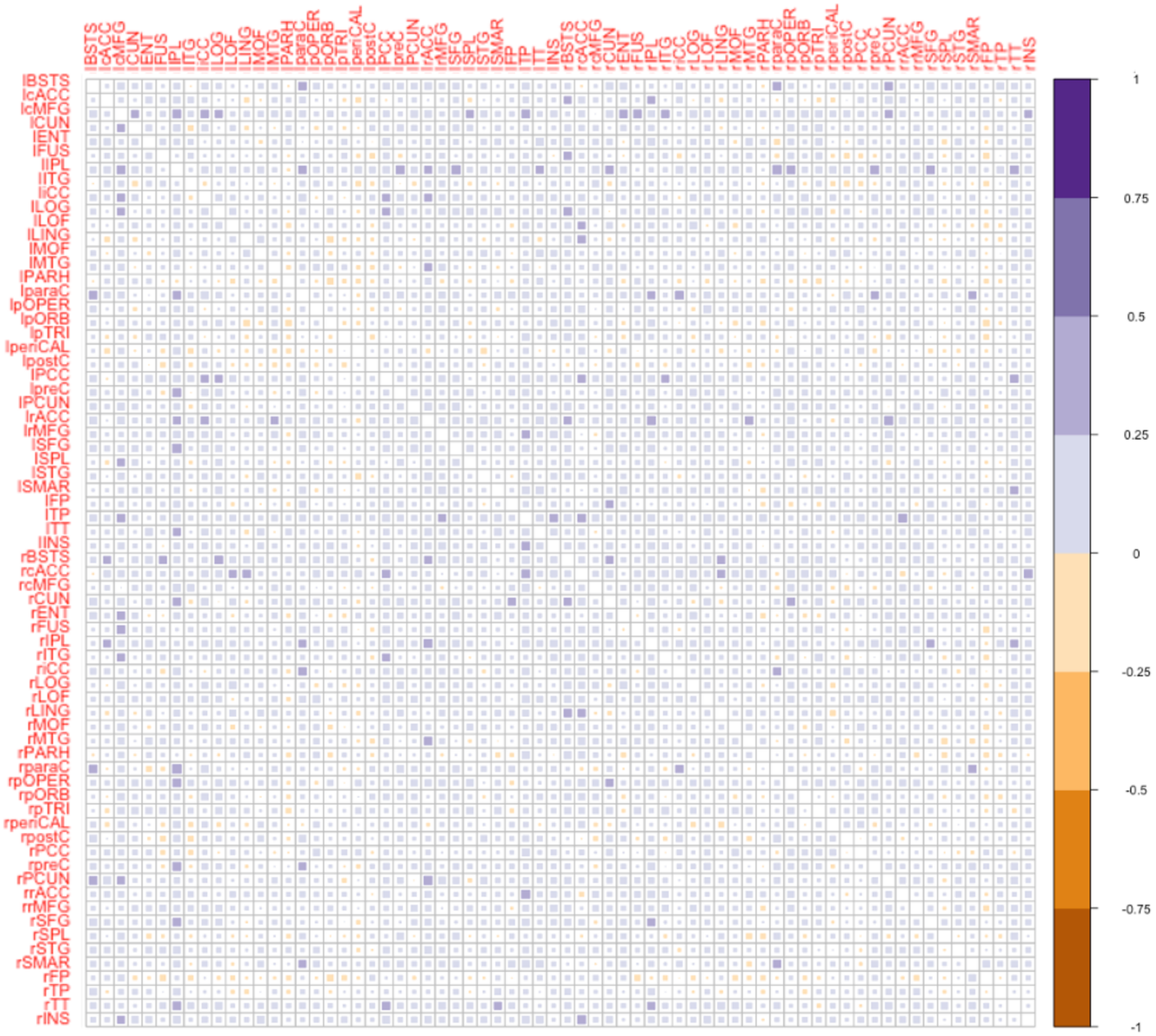

Supplementary Figure 6. *Correlogram showing the difference in inter-regional correlation coefficients in PD–VH vs PD-noVH patients for cortical surface area. The orange squares indicate inter-regional coefficients that were greater for PD-noVH.*

Cortical Thickness

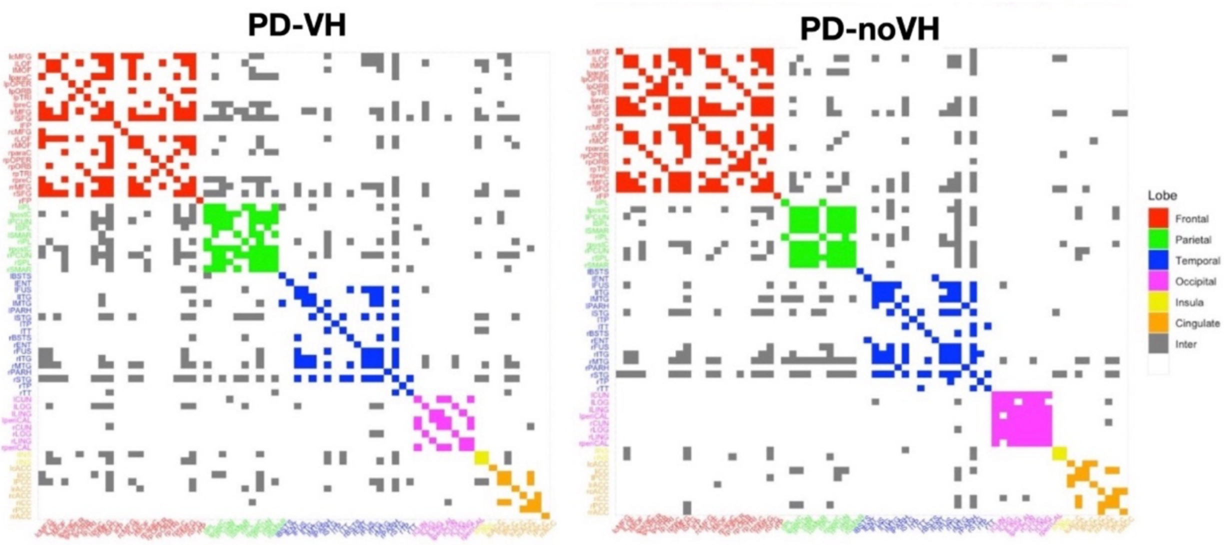

Surface area

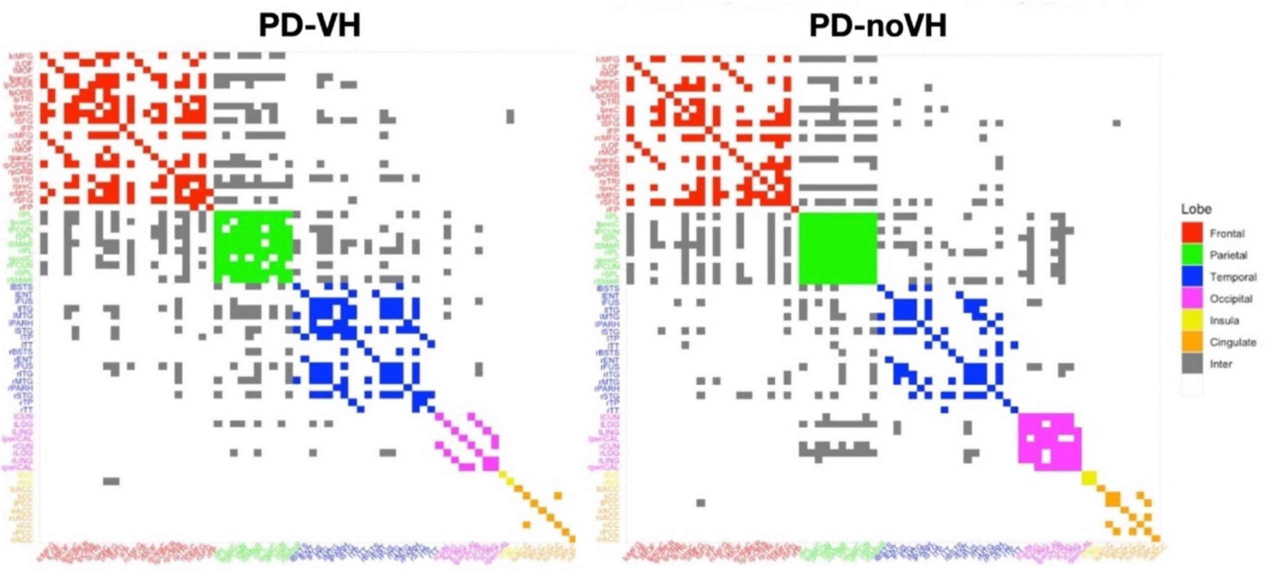

Supplementary Figure 7. *Community plot by lobe for cortical thickness (top row), surface area (bottom row). Legend: Red = frontal: bilateral caudal middle frontal gyrus, lateral orbital frontal gyrus, middle orbital frontal gyrus, paracentral gyrus, inferior frontal gyrus orbital, opercularis, triangular, precentral gyrus, middle frontal gyrus, superior frontal gyrus, frontopolar. Green = parietal: bilateral inferior parietal lobe, postcentral gyrus, precuneus, superior parietal lobe, supra marginal gyrus. Blue = temporal: banks superior temporal, enthorinal, fusiform gyrus, inferior temporal gyrus, middle temporal gyrus, parahippocampal gyrus, superior temporal gyrus,temporoparietal, temporal transverse. Pink = occipital: cuneus, lateral occipital gyrus, lingual gyrus, pericallosal. Yellow = Insula. Orange = cingulate: anter*

b) In order to further support our suggestion of particular networks involved, mentioned in the Discussion, we have also used, from the covariance analysis sample, the regions considered to be part of the DAN, VAN and DMN. We have compared the means of thickness and area in such regions in noVH vs VH (with age and gender as covariates). For this purpose we have collated together the mean of the regions making up each network and compared patients on those. These are the regions we used, based on Shine et al., 2014, 2015 (see References).

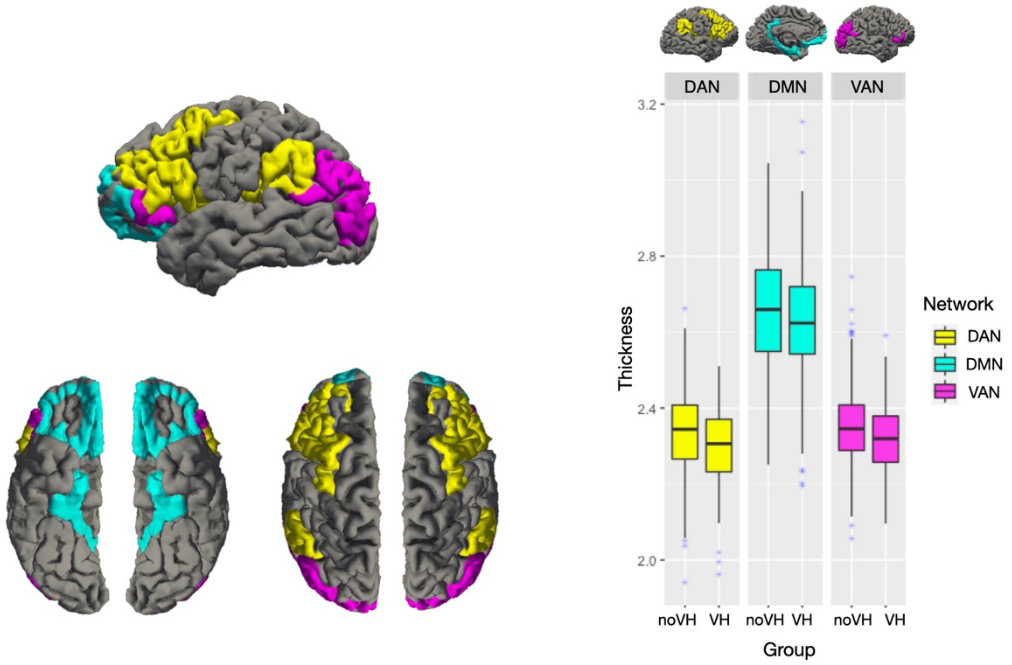

Supplementary Figure 7. *Brain render showing the regions selected for the networks and bar plots showing the results, where we find significant differences in the VAN and the DAN, but not for the DMN.*

DAN
Dorsolateral PFC – IFG opercularis and triangularis
Posterior parietal cortex - supramarginal gyrus
Frontal eye fields – precentral gyrus
Corpus striatum – this was not available from the current analyses

VAN
Basolateral amygdala - amygdala – this was not included in this analysis as the measurement is different for subcortical volumes than for thickness and area. However differences in the amygdala were reported here for both the main and the NPI sample.
Lateral and inferior PFC – IFG orbitalis, lateral orbitofrontal cortex (lOFC)
Temporoparietal junction - used the inferior parietal lobule (IPL)

Ventral striatum – as for the amygdala, we did not include the accumbens in this analysis, but we had it in the main analysis and no difference was observed in this region.

DMN
MT – PHG and enthorinal
Medial PFC - medial orbitofrontal cortex (mOFC) and frontopolar region
Posterior cingulate cortex

For thickness we find significant differences in the VAN [F(3,462) =9.45, *p*= .002, partial eta squared = 0.02, CI 0.01;0.05)] and the DAN [F(3,462) =12.46, *p* < .001, partial eta squared = 0.03, 0.02;0.06)] being reduced in thickness in PD-VH, but not in the DMN [F(3,462) =1.87, *p*= .17, partial eta squared = 0.004, CI -0.01;0.05)], whereas we do not

find any difference for surface area. This result is consistent with our suggestion that the group differences particularly involve the dorsal and ventral attention networks. In addition, both results are consistent with the differences found in the single regions making up such network in the main sample analysis.

Nevertheless, this analysis mainly summarises structural differences possibly related to neural pathology in these networks, whereas the findings of the structural covariance analysis investigate network-level properties related to cortical thinning and anomalies in surface area taking into account not only morphometric differences but connectivity metrics.
