# Supplementary material for: Mapping brain structural differences and neuroreceptor correlates in Parkinson’s disease visual hallucinations
Source: Nat Commun. 2022 Jan 26;13:519. doi: 10.1038/s41467-022-28087-0 (PMC8791961; doi:10.1038/s41467-022-28087-0)
Supplement: Supplementary file 3 — Reporting Summary [file 41467_2022_28087_MOESM3_ESM.pdf]

nature research

Corresponding author(s): Miriam Vignando

Last updated by author(s): Nov 24, 2021

Reporting Summary

Nature Research wishes to improve the reproducibility of the work that we publish. This form provides structure for consistency and transparency in reporting. For further information on Nature Research policies, see Authors & Referees and the Editorial Policy Checklist.

Statistics

For all statistical analyses, confirm that the following items are present in the figure legend, table legend, main text, or Methods section.

n/a	Confirmed	
☐	☒	The exact sample size (n) for each experimental group/condition, given as a discrete number and unit of measurement
☐	☒	A statement on whether measurements were taken from distinct samples or whether the same sample was measured repeatedly
☐	☒	The statistical test(s) used AND whether they are one- or two-sided<br>*Only common tests should be described solely by name; describe more complex techniques in the Methods section.*
☐	☒	A description of all covariates tested
☐	☒	A description of any assumptions or corrections, such as tests of normality and adjustment for multiple comparisons
☐	☒	A full description of the statistical parameters including central tendency (e.g. means) or other basic estimates (e.g. regression coefficient) AND variation (e.g. standard deviation) or associated estimates of uncertainty (e.g. confidence intervals)
☐	☒	For null hypothesis testing, the test statistic (e.g. F, t, r) with confidence intervals, effect sizes, degrees of freedom and P value noted<br>*Give P values as exact values whenever suitable.*
☐	☒	For Bayesian analysis, information on the choice of priors and Markov chain Monte Carlo settings
☐	☒	For hierarchical and complex designs, identification of the appropriate level for tests and full reporting of outcomes
☐	☒	Estimates of effect sizes (e.g. Cohen's d, Pearson's r), indicating how they were calculated

Our web collection on statistics for biologists contains articles on many of the points above.

Software and code

Policy information about availability of computer code

Data collection	No software used for data collection.
Data analysis	SPSS 24.0.0, R 4.0.0, braingraph 2.7.0, factominer 2.4, factoextra 1.0.7 igraph 1.2.7, rstatix 0.7.0, Freesurfer 6.0.0, MASS 7.3-54, ggplot2 3.3.5

For manuscripts utilizing custom algorithms or software that are central to the research but not yet described in published literature, software must be made available to editors/reviewers. We strongly encourage code deposition in a community repository (e.g. GitHub). See the Nature Research guidelines for submitting code & software for further information.

Data

Policy information about availability of data

All manuscripts must include a data availability statement. This statement should provide the following information, where applicable:

- Accession codes, unique identifiers, or web links for publicly available datasets
- A list of figures that have associated raw data
- A description of any restrictions on data availability

Data availability: Pre-harmonisation and post-harmonisation summary data, the [18F] Fallypride template are available with this publication. The 5-HT maps can be found at: https://xtra.nru.dk/FS5ht-atlas/. Further information and request for resources should be directed to and will be fulfilled by the lead author Miriam Vignando (miriam.vignando@kcl.ac.uk). This study did not generate new unique reagents.

Code availability: We did not generate new ad hoc code for the study, as all the analyses were based on pre-existing R packages or publicly available codes. We provide the R codes generated for the structural covariance analysis (R package braingraph 2.7.0), for the principal component analysis (R package factominer 2.4) and the receptor density maps models (R package rstatix 0.7.0 (and MASS 7.3-54).

Field-specific reporting

Please select the one below that is the best fit for your research. If you are not sure, read the appropriate sections before making your selection.

☒ Life sciences ☐ Behavioural & social sciences ☐ Ecological, evolutionary & environmental sciences

For a reference copy of the document with all sections, see nature.com/documents/nr-reporting-summary-flat.pdf

Life sciences study design

All studies must disclose on these points even when the disclosure is negative.

Sample size	Pre-collected data was used in the study. Our sample size is N=493, to ensure enough power to detect differences between groups and to perform structural covariance analyses
Data exclusions	N=20 total participants who did not meet the criteria in terms of diagnosis (e.g. healthy controls,with diagnosis of dementia) or whose scan did not segment well during pre-processing (motion) (N=6) were discarded.
Replication	We performed 3 replication analyses: 1) subsample of 440 participants with additional covariates (S3b), 2) subsample of 146 participants with additional covariates (S4) (the subsample underwent also additional analyses reported in the main text), 3) 'leave one group out' approach in 3c. Results support the findings of the main analysis.
Randomization	As we used data pre.collected by other groups, participants were originally allocated to the 'non hallucinations' or 'hallucinations' groups based on questionnaire (neuropsychiatric inventory, NEVHI, UPDRS) and clinical evaluation. We reatained the original allocations.
Blinding	The study is an analysis of structural brain differences in patients with and without hallucinations on pre-collected data, blinidng was not applicable.

Reporting for specific materials, systems and methods

We require information from authors about some types of materials, experimental systems and methods used in many studies. Here, indicate whether each material, system or method listed is relevant to your study. If you are not sure if a list item applies to your research, read the appropriate section before selecting a response.

Materials & experimental systems

n/a	Involved in the study	
☒	☐	Antibodies
☒	☐	Eukaryotic cell lines
☒	☐	Palaeontology
☒	☐	Animals and other organisms
☐	☒	Human research participants
☒	☐	Clinical data

Methods

n/a	Involved in the study	
☒	☐	ChIP-seq
☒	☐	Flow cytometry
☐	☒	MRI-based neuroimaging

Human research participants

Policy information about studies involving human research participants

Population characteristics	The final sample comprised 493 participants, 135 with VH (62F , age = 67.85, SD = 7.74), 358 (131 F, age = 65.66, SD = 8.71) without VH.
Recruitment	We used pre-collected data from other groups that was subsequently transferred to KCL, thus we did not recruit any participant for the study.
Ethics oversight	The study obtained King's College London ethical approval from Research Ethics Office, Psychiatry, Nursing and Midwifery (PNM) Research Ethics Panel (LRS-19/20-17680) on the 25/03/2020 and is pre-registered on the Open Science Framework site on 04/05/2020 (https://osf.io/nzatk).

Note that full information on the approval of the study protocol must also be provided in the manuscript.

Magnetic resonance imaging

Experimental design

Design type	Structural T1-weighted multi-site harmonisation mega analysis.
Design specifications	Group-level analysis (between subjects MANCOVA, between subjects factor: visual hallucinations vs. no hallucinations). Receptor density maps regression models. Structural covariance. Principal component analysis.
Behavioral performance measures	Hallucination status, NPI score.

Acquisition

Imaging type(s)	Structural.
Field strength	3T and 1.5T.
Sequence & imaging parameters	This is a multi-site study, every site had different scanners and acquisition protocols.<br>Data was harmonized with Combat for multi-site and scanner effects.
Area of acquisition	Brain

Diffusion MRI ☐ Used ☒ Not used

Preprocessing

Preprocessing software	Freesurfer 6.0.0 standard recon-all procedure, consisting of motion correction, skull-stripping, affine registration to Talairach atlas, segmentation, smoothing, and parcellation mapping.
Normalization	Data was Talairach transformed and normalized with the standard normalization step in the recon-all pipeline
Normalization template	fsaverage
Noise and artifact removal	Motion and intensity correction were applied prior normalization and skull striping. Then standard recon-all pipeline was executed. At the end of the process, in order to screen for possible errors in the segmentation process, mean cortical thickness measures and manual slice by slice inspection were used to identify possible errors in the white-grey matter boundary and pial reconstruction steps. For subjects that did not segment properly the failed processing steps were re-run (after performing the appropriate corrections. Low quality scans<br>(e.g. with excessive motion, n= 4) or scans that did not segment well upon troubleshooting (n =2) were discarded
Volume censoring	Standard motion correction was applied (Freesurfer recon-all pipeline)

Statistical modeling & inference

Model type and settings	Group-level analyses: Multivariate analysis of covariance (age, gender, TIV as covariates, hallucinaions present/not present as between subjects factor); sensitivity analysis<br>on subsample, multivariate analysis of covariance (Age, gender, TIV, onset, medication, cognition, PD severity); s tructural covariance (age, gender as covariates); regression models with receptor density maps and correction for spatial autocorrelation; principal component analysis; partial correlations analyses
Effect(s) tested	Hallucinations vs. no hallucinations, tested with Multivariate analysis of covariance (age, gender, TIV as covariates, hallucinaions present/not present as between subjects factor) and the models described in the box above.

Specify type of analysis: ☐ Whole brain ☒ ROI-based ☐ Both

Anatomical location(s)	We used the Destrieux and Desikan-Killiany atlases. All ROIs were included in the analyses.
Statistic type for inference<br>(See Eklund et al. 2016)	Vertex-wise
Correction	FDR.

Models & analysis

n/a	Involved in the study
☒	☐ Functional and/or effective connectivity
☐	☒ Graph analysis
☒	☐ Multivariate modeling or predictive analysis

Graph analysis

Dependent variable: hallucinations yes/no. Binarised graph, group level. Vertex importance was assessed using degree, betweenness centrality and nodal efficiency. A hub was categorised as such if its betweenness centrality was greater than the mean plus 1 standard deviation - calculated on all regions at the same density. Analyses on efficiency.
